# Organization of Physical Interactomes as Uncovered by Network Schemas

**DOI:** 10.1371/journal.pcbi.1000203

**Published:** 2008-10-24

**Authors:** Eric Banks, Elena Nabieva, Bernard Chazelle, Mona Singh

**Affiliations:** Department of Computer Science & Lewis-Sigler Institute for Integrative Genomics, Princeton University, Princeton, New Jersey, United States of America; Columbia University, United States of America

## Abstract

Large-scale protein-protein interaction networks provide new opportunities for understanding cellular organization and functioning. We introduce *network schemas* to elucidate shared mechanisms within interactomes. Network schemas specify descriptions of proteins and the topology of interactions among them. We develop algorithms for systematically uncovering recurring, over-represented schemas in physical interaction networks. We apply our methods to the *S. cerevisiae* interactome, focusing on schemas consisting of proteins described via sequence motifs and molecular function annotations and interacting with one another in one of four basic network topologies. We identify hundreds of recurring and over-represented network schemas of various complexity, and demonstrate via graph-theoretic representations how more complex schemas are organized in terms of their lower-order constituents. The uncovered schemas span a wide range of cellular activities, with many signaling and transport related higher-order schemas. We establish the functional importance of the schemas by showing that they correspond to functionally cohesive sets of proteins, are enriched in the frequency with which they have instances in the *H. sapiens* interactome, and are useful for predicting protein function. Our findings suggest that network schemas are a powerful paradigm for organizing, interrogating, and annotating cellular networks.

## Author Summary

Large-scale networks of protein-protein interactions provide a view into the workings of the cell. However, these interaction maps do not come with a key for interpreting them, so it is necessary to develop methods that shed light on their functioning and organization. We propose the language of network schemas for describing recurring patterns of specific types of proteins and their interactions. That is, network schemas describe proteins and specify the topology of interactions among them. A single network schema can describe, for example, a common template that underlies several distinct cellular pathways, such as signaling pathways. We develop a computational methodology for identifying network schemas that are recurrent and over-represented in the network, even given the distributions of their constituent components. We apply this methodology to the physical interaction network in *S. cerevisiae* and begin to build a hierarchy of schemas starting with the four simplest topologies. We validate the biological relevance of the schemas that we find, discuss the insights our findings lend into the organization of interactomes, touch upon cross-genomic aspects of schema analysis, and show how to use schemas to annotate uncharacterized protein families.

## Introduction

Recent high-throughput experimental methods have generated proteome-scale protein-protein physical interaction maps for many organisms (review, [1]). Computational analyses of these networks have identified global topological and dynamic features [2],[3] and have revealed a modular organization [4] with highly connected groups of proteins taking part in the same biological process or protein complex [5],[6]. Further analysis has shown that the wiring diagrams of biological networks are comprised of network motifs, or particular circuits, that occur more frequently than expected by chance [7]–[13].

**Figure 1:**
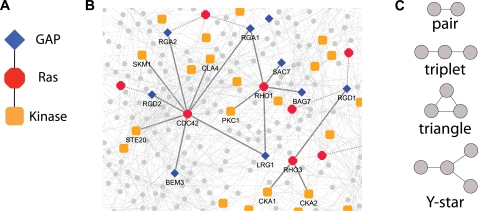
Network schemas: an example and the topologies considered. (A) An example of a triplet schema. Ras signaling involves a small G protein from the Ras family, which is regulated by a GTPase Activating Protein (GAP) and in turn regulates its effector kinase. The corresponding *GAP-Ras-Kinase* schema has a Ras protein interacting with a GAP protein as well as a kinase. (B) Instances of the *GAP-Ras-Kinase* schema in the *S. cerevisiae* physical interactome. Only a portion of the yeast physical protein-protein interaction network is shown. Ras family proteins are displayed as red octagons, GAP proteins as blue diamonds, and kinases as orange squares. Interactions that comprise an instance of a *GAP-Ras-Kinase* triplet schema are illustrated with thick solid lines, while other GAP-Ras and Ras-kinase interactions are marked by thick dashed lines. See Methods for construction of physical interaction network and determination of protein annotations. This and subsequent figures are created using Cytoscape [74]. (C) Schema topologies that are considered in this study.

We advocate an orthogonal view of network make-up whereby organizational units consist of specific, and potentially different, types of proteins that preferentially work together in various network topologies. Thus, we aim to explicitly incorporate known attributes of individual proteins into the analysis of biological networks. We conceptualize this with *network schemas*, which are a general means for representing organizational patterns within interactomes where groups of proteins are described by arbitrary known characteristics along with the desired network topology of interactions among them (Figure 1A). A schema's matches (or instances) in an interactome are subgraphs of the interaction network that are made up of proteins having the specified characteristics which interact with one another as dictated by the schema's topology (Figure 1B). For example, a schema associated with signaling might be a linear path of kinases interacting in succession; its instances in *S. cerevisiae* include portions of the pheromone response and filamentous growth pathways. Although any property can be used to annotate proteins in schemas, and different types of interactions may be specified, we focus on direct physical protein-protein interactions with proteins described via Pfam sequence motifs [14] and a set of GO molecular function terms [15]; such schemas with multiple instances in an interactome are likely to correspond to shared mechanisms that underlie a range of biological activities. Because we expect the largest number of schemas with multiple instances to be associated with small topologies, we begin to address these questions by considering four basic network topologies (Figure 1C) varying from two interacting proteins (pair schemas) to higher-order schemas containing up to three interactions (triplet, triangle, and Y-star schemas); we choose these particular linear, cyclical, and branched topologies because they are the simplest patterns in physical interactomes that may intuitively be associated with signaling pathways, complexes, and switch-like patterns, respectively.

This paper has three major contributions. First, we develop a computational procedure for automatically identifying *emergent* network schemas, or schemas that are both recurrent and over-represented in the interactome even when the frequencies of their lower-order subschemas are considered. Conditioning over-representation on the distribution of a schema's lower-order constituents ensures that every emergent schema conveys novel information about interactome organization. We score a schema based upon its frequency in the interaction network and its expected frequency given the distribution of its constituent subschemas. The expected frequency is computed using a carefully designed graph randomization algorithm that preserves the distributions of the specific labeled subschemas. The false discovery rate of the resulting scores is then evaluated using a variant of the permutation test. We note that in order to uncover emergent schemas, existing approaches for related problems could not be directly utilized; the specifics and scale of this problem required the development of novel computational techniques (see **Methods** for more details).

Second, in the first large-scale analysis of this type, we apply our procedure to the *S. cerevisiae* protein-protein interactome. In total, more than 140,000 Pfam network schemas that occur at least once in the *S. cerevisiae* interactome are considered. Of these, we identify 264 emergent Pfam network schemas with various annotations and topologies. We also uncover 138 emergent GO molecular function pair schemas. Analysis of emergent network schemas reveals a network organization where pair schemas are most diverse and where higher-order schemas reveal complex networks of primarily signaling and transport related activities. This suggests that the recurring units within interactomes are mostly pairwise, but that for some functions, higher-order recurring units are still prevalent. The hierarchical nature of emergent schemas can be visualized in a graph-theoretic manner which highlights that certain lower-order schemas occur frequently in higher-order emergent schemas (i.e., they are “hubs” in these networks), even though the frequencies of the lower-order schemas are controlled for in the computational procedure.

Third, we demonstrate that emergent network schemas correspond to biologically meaningful units. In particular, in a systematic analysis, we show that schema instances lead to protein subnetworks that share more specific biological process annotations than subnetworks having identical topologies but no constraints on the proteins making them up; this illustrates the additional benefit of incorporating protein annotations into traditional topology-based network analysis. Moreover, at the other extreme of the eukaryotic spectrum, we find that if we interrogate the *H. sapiens* interactome using the emergent schemas uncovered in *S. cerevisiae*, more than one-half of the schemas of each topology have instances there as well; this fraction is considerably lower when considering non-emergent *S. cerevisiae* schemas. Finally, we give a proof of concept through two uncharacterized protein families that network schemas can be used to functionally characterize protein families and individual proteins.

### Relationship to previous work

Network schemas build upon earlier pioneering work in network analysis by enabling new types of analyses that were not possible with previous methods for identifying recurrent patterns in biological networks. By considering the specific roles of individual proteins, network schemas look beyond the purely topological features that are described by network motifs [7]–[13],[16] to the tendency of certain types of proteins to work together, thereby shifting focus from the “syntax” of biological networks to their “semantics.” While from a graph-theoretic point of view one may think of network schemas as a generalization of network motifs, considering protein attributes fundamentally changes what types of biological questions can (or cannot) be answered, and the much larger number of schemas changes the underlying computational issues as well. As compared to network alignments that uncover conserved interactions among homologous proteins in interactomes (e.g., [17]–[20]), network schemas utilize more abstract descriptions of proteins and are identified via a statistical model designed to find a hierarchy of interactome organizational units of increasing complexity. In contrast to approaches to uncover correlated sequence-signatures or putative domain-domain or domain-peptide interactions via analysis of interactomes (e.g., [21]–[31]), network schemas incorporate higher-order topologies. Moreover, unlike the approaches that particularly focus on identifying domain-domain or domain-peptide interactions, schemas do not focus on the physical bases for protein interactions. Therefore, they represent more abstract organizational units, indicating what types of proteins work together and not which portions of the protein are responsible for the observed interactions. Further, it is important to note that combinations of pair schemas present in the interactome result in higher-order schemas that do not necessarily occur, and thus it is necessary to explicitly enumerate over these in order to uncover which exist in the interactome. Compared to a very recent approach for uncovering over-represented functional attributes in linear paths in regulatory networks [32], network schemas additionally consider cyclical and branched schema topologies, and their relationships to lower-order schemas. Finally, as opposed to a number of approaches for finding the instances of particular (user-supplied) labeled subgraphs, which we term schemas, within a wide range of biological networks [32]–[38], our goal is to determine automatically which schemas are frequent and over-represented, and thus interesting enough to merit further analysis.

## Results

### Emergent network schemas in the *S. cerevisiae* interactome

Each pair schema is scored by considering its number of occurrences in the *S. cerevisiae* interactome against its average number of occurrences in degree-preserving random networks [7],[8],[39]. Each triplet, triangle, and Y-star schema is scored similarly, except that its average number of occurrences is computed in networks randomized so as to maintain the distribution of its constituent pairs (for triplet and triangle schemas) or its constituent triplets (for Y-star schemas). Using a false discovery rate (FDR) of ≤0.05, we identify 151 pair, 55 triplet, 26 triangle, and 32 Y-star Pfam emergent schemas in the *S. cerevisiae* network comprised of direct physical interactions. The emergent schemas are a small fraction of the total number of schemas occuring in the interactome. In total, 2838 pair, 24662 triplet, 999 triangle and 114650 Y-star Pfam schemas occur at least once in the *S. cerevisiae* interactome. Of these, 419 pair, 842 triplet, 31 triangle, and 999 Y-star schemas are recurring in that they have at least two non-overlapping instances (i.e., that do not contain a protein in common). All emergent schemas and supporting information are listed in Tables S1, S2, S3, S4, S5, S6, S7, S8, S9, S10, S11 and S12, including their FDRs, their average number of instances in the randomized networks, and their instances in *S. cerevisiae*.

**Figure 2:**
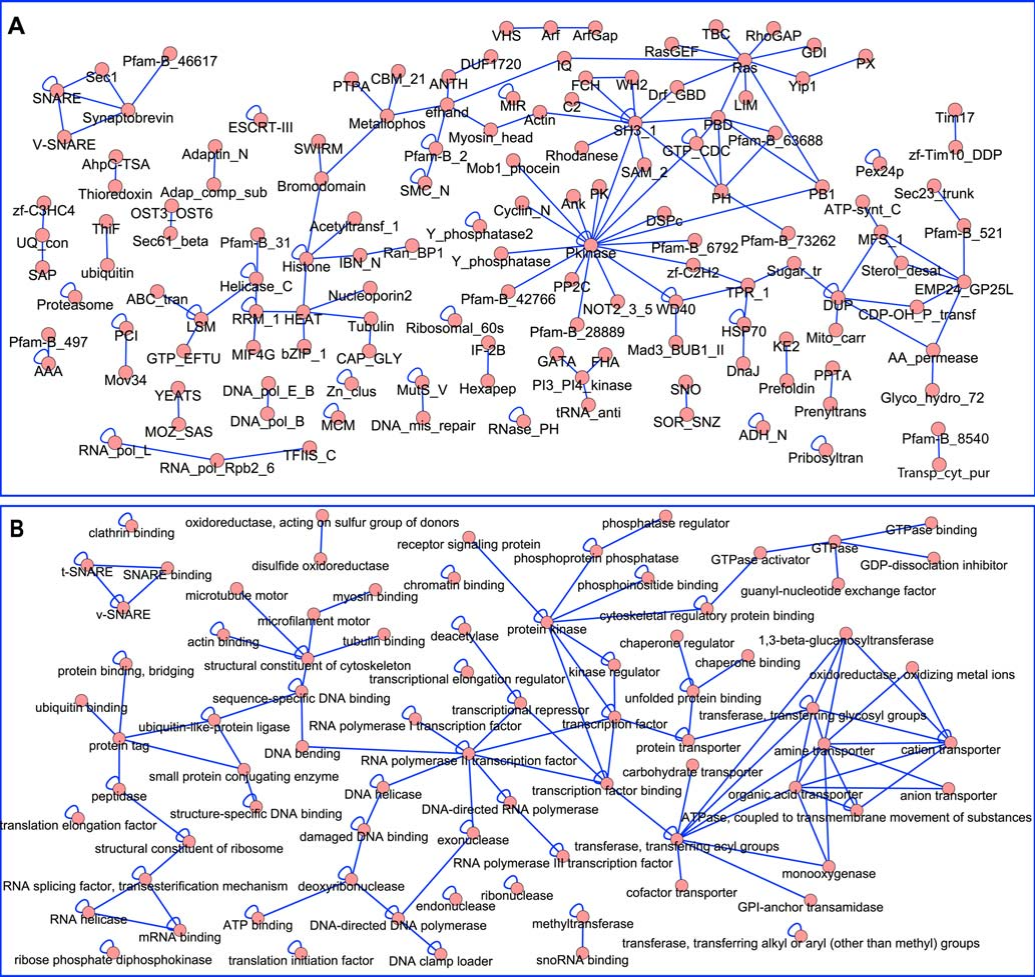
Emergent pair schemas uncovered in the *S. cerevisiae* interactome. A pair of vertices connected by an edge corresponds to a pair schema. (A) Pfam emergent pair schemas, where each vertex is labeled with a Pfam motif. (B) Gene Ontology molecular function emergent pair schemas, where each vertex is labeled with a GO molecular function term, with the word “activity” dropped from term names. See also Tables S1, S2 and S6.

The emergent pair schemas are depicted in a network in Figure 2A. Pair schemas represent two proteins working together (as a dimer or as part of a complex), or one protein (de)activating another. The uncovered emergent schemas represent a wide variety of functions including signaling (e.g., schemas involving *Pkinase* or *Ras* motifs), transport (e.g., schemas involving the amino acid permease motif *AA_permease*), intracellular trafficking (e.g., *synaptobrevin* schemas), RNA processes (e.g., *RRM_1* schemas) and ubiquitination (e.g., ubiquitin-conjugating enzyme motif *UQ_con* schemas). While some of the pair schemas may correspond to actual domain-domain interactions, the schema formulation by itself does not make any claims about the interaction interface. In particular, some of the underlying physical interactions may instead consist of domains interacting with peptides or disordered regions [40]. This is clear, for example, when looking at the diverse set of pair schemas involving the *SH3* domain which is known to typically bind proline-rich peptides [41]. Nevertheless, similar to earlier findings for domain-domain interactions [31], we find that emergent Pfam pair schemas are enriched in homotypic annotations as compared to all Pfam pair schemas in the interactome (18.5% vs. 5.8%).

We also uncover *S. cerevisiae* emergent pair schemas using a hand-chosen set of GO molecular function annotations (Figure 2B and Tables S1 and S6). As with the Pfam schemas, the GO pair schemas represent many types of functions including transport, signaling, DNA and RNA processing, ubiquitination, protein folding, and cytoskeleton organization. The GO molecular function schemas can sometimes allow generalizations of the Pfam schemas that move beyond sequence similarity, as proteins annotated with the same GO molecular function term need not be homologous to each other. For example, the Pfam pair schema consisting of a protein with the *Pkinase* motif interacting with a protein with the cyclin N-terminal motif *Cyclin_N* is subsumed by the GO schema consisting of a protein with kinase activity interacting with a protein with kinase regulator activity. Instances of this GO schema in the *S. cerevisiae* interactome include cyclins which lack the *Cyclin_N* Pfam motif, other cyclin-like proteins, and different kinase regulators altogether, such as activating subunits of kinase complexes, adaptors, and scaffold proteins. As another example, the Pfam pair schema consisting of the *Pkinase* motif interacting with the zinc finger motif *zf-C2H2* has a correspondence in a GO schema consisting of a protein with kinase activity interacting with a protein with transcription factor activity; instances of the latter schema in the *S. cerevisiae* interactome include transcription factors of the zinc finger, MADS, and basic helix-loop-helix families.

**Figure 3 pcbi-1000203-g003:**
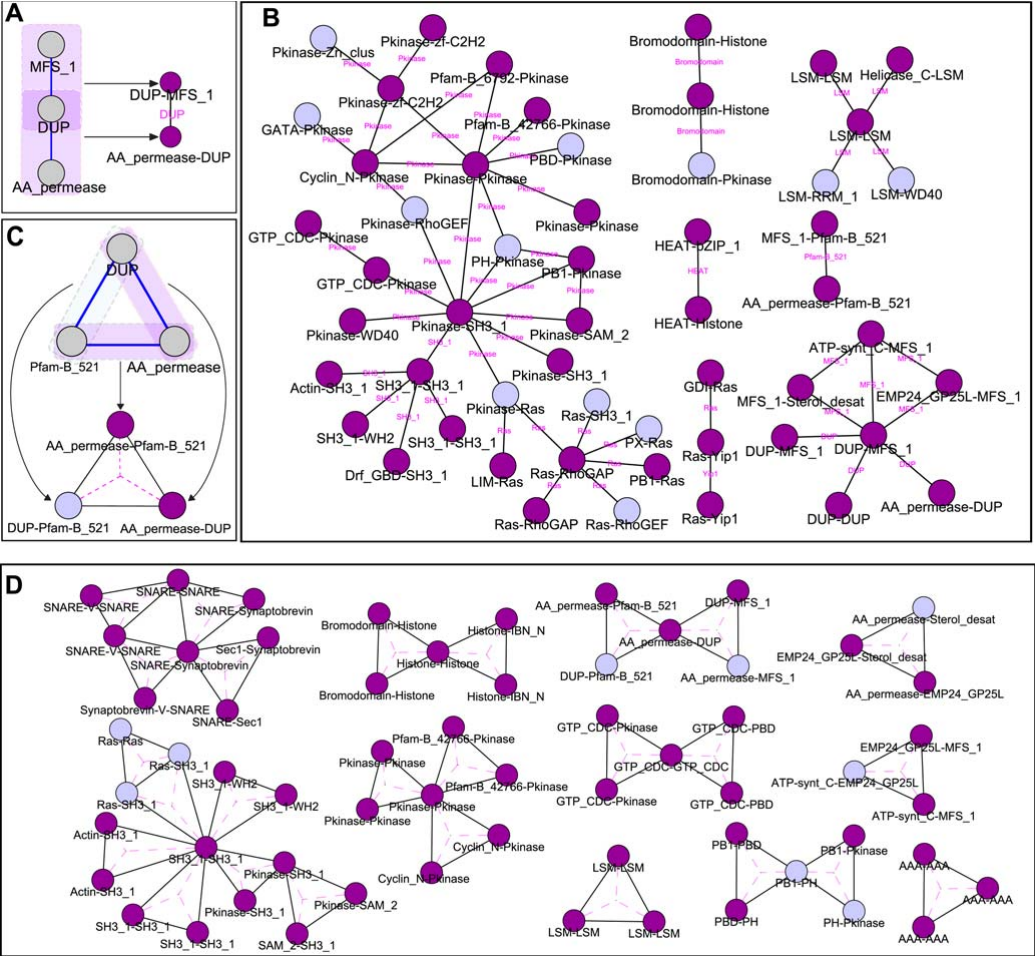
Emergent triplet and triangle schemas uncovered in the *S. cerevisiae* interactome, represented in a graph where vertices correspond to pair schemas. Pair schemas that are themselves emergent (Figure 2) are displayed as darker vertices. See also Tables S3 and S4. (A) An illustration of the subgraph representation for triplet schemas. The triplet *MFS_1-DUP-AA_permease* (on the left) is mapped to two pair vertices, corresponding to the lower-order pair schemas making it up, connected by an edge. The edge is labeled in pink with the central motif of the triplet (DUP). (B) Pfam emergent triplet schemas. (C) An illustration of the triangle schema *DUP-AA_permease-Pfam-B_521*. The triangle *DUP-AA_permease-Pfam-B_521* is mapped to three pair vertices, corresponding to the lower-order pair schemas making it up, connected by edges; that is, it is represented as a triangle in the graph whose vertices represent pair schemas. The *DUP-Pfam-B_521* pair, colored pale in the pair-vertex graph, is not an emergent pair schema, whereas the other two pairs in the triangle, colored dark in the pair-vertex graph, are. (D) Pfam emergent triangle schemas.

**Figure 4 pcbi-1000203-g004:**
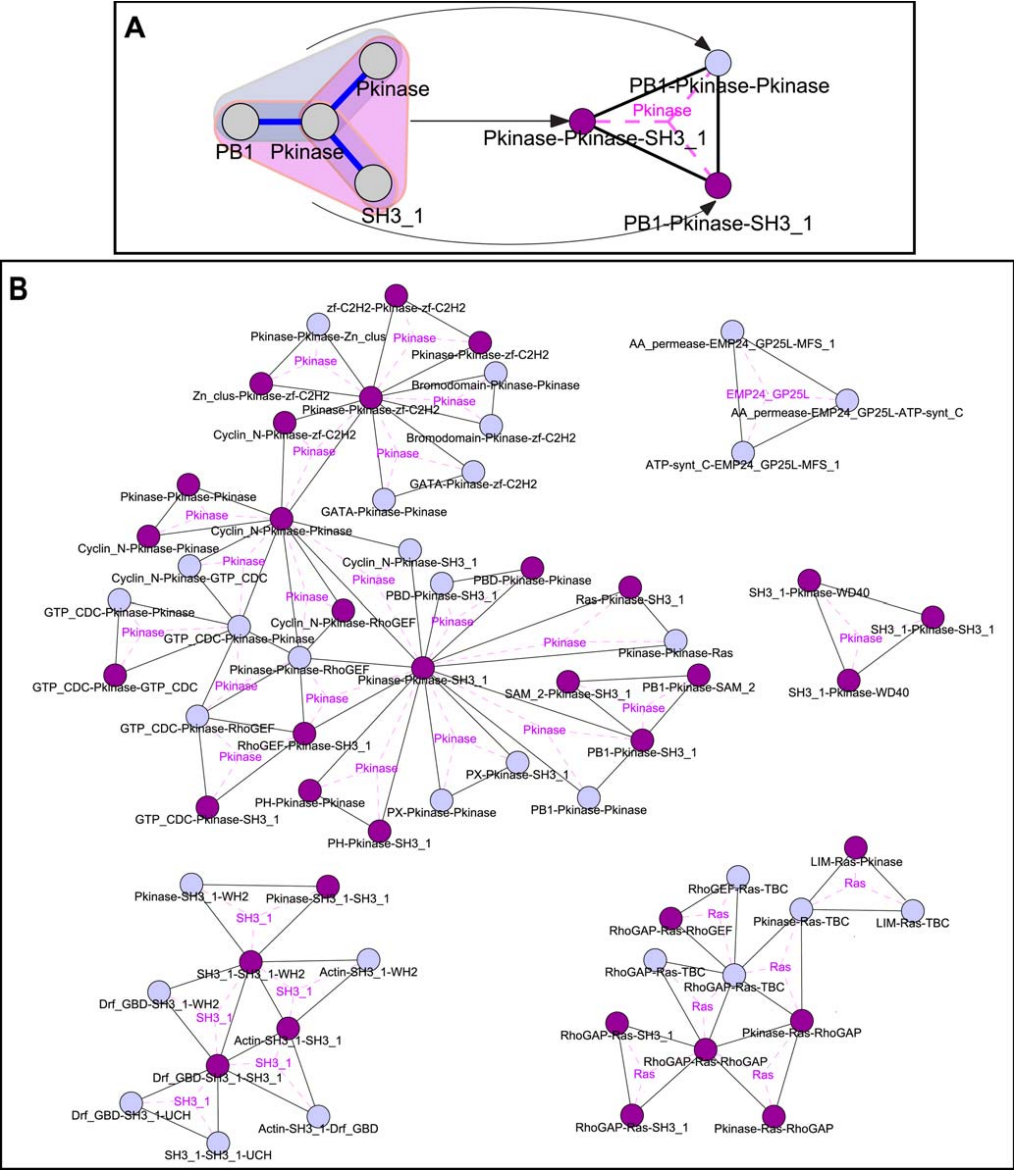
Emergent Y-star schemas uncovered in the *S. cerevisiae* interactome, represented as triangles in a graph where each vertex corresponds to a triplet schema. Triplet schemas that are themselves emergent (Figure 3) are displayed as darker vertices. (A) An illustration of the triplet subgraph representation of a Y-star schema. The Y-star (on the left) is mapped to three vertices corresponding to its lower-order triplet schemas, along with edges among them; that is, it is represented as a triangle in the graph whose vertices represent triplet schemas. The triplet subschemas of the Y-star are highlighted. The subschemas that are emergent triplets are highlighted in purple and represented as darker vertices. For ease of visualization, the central node of the Y-star is labeled in pink inside the triangle and connected to the vertices by dashed lines. (B) Pfam emergent Y-star schemas. See also Table S5.

Higher-order emergent *S. cerevisiae* network schemas are given in Figures 3 and 4. For the purpose of visualization, they are represented as networks where vertices correspond to lower-order schemas. That is, for each higher-order schema, there is a vertex for each of its corresponding lower-order schemas, along with edges between these vertices; triplets and triangles are depicted with respect to lower-order pair schemas whereas Y-stars are depicted with respect to lower-order triplet schemas (see Figures 3A, 3C, and 4A for explanation). Edges in these networks thereby indicate that the two corresponding lower-order schemas are found together as parts of a emergent higher-order schema.

The uncovered emergent triplet schemas (Figure 3B) include several relating to signaling (e.g., *Pkinase* and *Ras* schemas) and transport (the connected components with the *MFS_1* motif). The signaling schemas include kinase cascades (e.g., *Pkinase*-*Pkinase*-*Pkinase*), regulation of Ras signaling (e.g., *RhoGAP*-*Ras*-*RhoGEF*), those connecting Ras and kinase signaling (e.g., *RhoGAP*-*Ras*-*Pkinase*), and those relating to specific structural domains involved in signaling [42] (e.g., *SH3*-*Pkinase*-*WD40* and *SH3*- *Pkinase*- *PH*). Note that there are many possible schemas associated with signaling (e.g., consider the set of schemas annotated with all domains known to be associated with signaling [42]), and our schema analysis identifies only a small subset of these as emergent. There are numerous emergent triplet schemas involving the major facilitator superfamily (*MFS_1*), one of the two largest families of membrane transporters [43]. Triplet *MFS_1* schemas include those involving other transport proteins, such as membrane proteins involved in transport of amino acids (i.e., containing the *AA_permease* motif) and proteins involved in ER to Golgi transport (e.g., containing the *EMP24* motif). Whereas the pervasiveness of kinases within conserved portions of the interactome has been observed earlier [17], the prevalence of such transport related subnetworks has been previously underappreciated.

Many of the triangle schemas (Figure 3D) correspond to known complexes. There are several triangle schemas, making up a connected component, corresponding to the SNARE vesicle-fusion machinery. The triangle schema made up of *LSM* motifs corresponds to Sm and LSM complexes, and is associated with the spliceosome as well as other RNA processing [44]. The triangle schema made up of *AAA* motifs corresponds to replication factor C complex and the 19S particle of the 26S proteosome. There are numerous triangle schemas associated with signaling as well; these may correspond, for example, to complexes or phosphorylation by kinase complexes. For example, the *Cyclin_N*-*Pkinase*-*Pkinase* triangle schema contains instances where a cyclin associates with a cyclin-dependent kinase, and this complex either phosphorylates or is phosphorylated by another kinase.

The emergent Y-star schemas (Figure 4B) refine the functional landscape of the triplet schemas, with one relating to transport and several relating to *Ras* and kinase signaling pathways. The Y-star schemas showcase the complex, nonlinear regulatory patterns evident in biological pathways. For example, some of the Y-star *Pkinase* schemas relate to the role of phosphorylation in combinatorial regulation of transcription factors (e.g., those including multiple transcription factor motifs, such as *zf-C2H2* and *GATA* interacting with the same kinase), whereas others correspond to kinase cascades that additionally incorporate regulation via cyclins (e.g., schemas including *Cyclin_N*). Additionally, several Y-star schemas represent a dynamic “switch-like” pattern in which the peripheral proteins are active in different contexts. This is evident in some schemas where the peripheral proteins belong to the same family, and utilize the same structural interface on the central protein. For example, several of the Y-star Ras schemas consist of a central Ras protein interacting with several regulatory GTPase activating proteins (corresponding to *RhoGAP*, *TBC* or some *LIM* containing proteins). Such schemas show that certain types of “mutually exclusive” interactions [45] recur together in the interactome.

### Schemas give insight into organizational principles of interactomes

While each emergent network schema represents a specific way in which proteins can work together, their relationships to one another, and in particular of higher-order schemas to lower-order ones, lead to some general observations about network organization.

The first observation is the striking drop in the number and diversity of emergent schemas with increased complexity, especially between pair and higher-order schemas (there are 139, 39, 29 and 30 distinct Pfam motifs involved in pair, triplet, triangle and Y-star emergent schemas respectively). Whereas 36% of the recurring pair schemas in *S. cerevisiae* are found to be emergent, only 6% of recurring triplet and 3% of recurring Y-star schemas are. (Note that triangle schemas are something of a special case because the cyclical structure is very constrained and recurring units are unlikely to be found at random.) This suggests that the semantic units within interactomes are primarily at the pair level, and that most repeated patterns of higher order can be viewed as rearrangements of the pairs that can be explained simply by randomness. At the same time, there are a considerable number of higher-order schemas (i.e., those identified as emergent) that cannot be explained by lower-order ones.

These higher-order emergent schemas are not just combinations of the lower-order emergent pair schemas. For example, the emergent pair schema network (Figure 2) contains 712 triplets, of which 571 occur even once in the *S. cerevisiae* interactome. Of these, only 37 are emergent. Thus, the majority of possible triplets resulting from emergent pair schemas are not emergent, and triplet schemas thereby allow us to uncover which sets of proteins comprising pair schemas work together in the network. On the other hand, 18 emergent triplet schemas are not present in the emergent pair schema network. For example, the *RhoGAP*-*Ras*-*Pkinase* emergent triplet schema consists of the *Ras*- *Pkinase* pair which is not found to be emergent. Though this pair occurs numerous times in the network, given the frequency of *Ras* and *Pkinase* Pfam motifs, it does not appear at the FDR≤0.05 level; this also demonstates that, as intended, our procedure for uncovering schemas corrects for the frequency of the motifs.

Large fractions of the distinct lower-order schemas making up the higher-order emergent schemas are themselves emergent (73% and 80% of the pair schemas comprising triplet and triangle schemas, respectively, and 51% of the triplet schemas making up Y-star schemas). The use of subgraph-preserving randomizations in our procedure confirms that this observation is not due solely to the abundance of the lower-order structures, but is a more general feature of schema organization. This result has a topological counterpart, as it has been found that four-protein network motifs tend to be combinations of three-protein ones [10].

Several emergent schemas from each topology share particular lower-order schemas. These lower-order schemas that are found in numerous higher-order schemas correspond to hubs in Figures 2, 3, and 4. We observe that the nodes with largest degree in the *S. cerevisiae* Pfam pair graph (Figure 2A) are *Pkinase*, *SH3_1*, and *Ras*. These domains comprise hubs at different levels of schema complexity. For example, the pairs that are hubs in the triplet graph (Figure 3B) are *Pkinase*- *SH3_1*, *Ras*-*RhoGAP*, and *Pkinase*-*Pkinase*. It is instructive to compare these families to the list of the 10 most frequent Pfam motifs and the 10 Pfam motifs involved in the highest number of interactions in the studied network (given in Table S8). As expected, because of our scoring procedure which considers the frequency of annotations in the network, while some of the “hub” motifs are frequent in the interactome or common in interactions (e.g., *Pkinase* and *SH3*), many are not (e.g., *RhoGAP*); additionally, there are many Pfam motifs that occur frequently in the network but are not prevalent in these schemas (e.g., *Helicase_C*).

### Schemas recapitulate known biology: the Ras superfamily

As an illustrative example showing that automatically uncovered emergent schemas can show excellent correspondence to well-understood organizational and functional units, we detail our findings on *S. cerevisiae* emergent Pfam schemas involving the Ras superfamily. There are ten *Ras* pair schemas (Figure 2A). The *Ras*-*RhoGAP*, *Ras*-*RasGEF*, and *Ras*-*TBC* schemas correspond to the basic regulatory interactions of Ras proteins. The *Ras*- *GDI* pair reflects the additional regulatory mechanism of the Rab subfamiliy of Ras proteins by the guanyl dissociation inhibitors (GDIs). The *Yip1* family of proteins in turn may act as GDI displacement factors [46] for a group of Ras-like proteins associated with Golgi membranes and/or act as membrane recruiters of these proteins [47]. Two Ras pair schemas involve Ras-binding motifs—the diaphanous GTPase-binding motif *DRF_GBD* found in Rho effectors and the P21-Rho-binding motif ( *PBD*). Other Ras pair schemas contain motifs that reflect the biological role of Ras families, such as the *IQ* calmodulin-binding motif and the *PB1* domain associated with signaling. Finally, *LIM* is a general structural domain, but is found in several GAP proteins. The higher-order Ras emergent schemas (Figures 3 and 4) include several that reflect their diverse regulatory mechanisms. For example, there is a *Pkinase*-*Ras*-*RhoGAP* triplet, where the *RhoGAP* regulates the *Ras* which in turn regulates the kinase, and a *RhoGEF*-*Ras*- *RhoGAP* triplet, where both the *RhoGEF* and *RhoGAP* regulate *Ras*.

### Schemas uncover functionally coherent portions of the interactome

To validate in a systematic manner that emergent schemas correspond to functional units and may be helpful towards uncovering network modularity, we determine whether individual instances of emergent schemas have enriched functional coherence beyond that suggested by guilt-by-association and subgraph topology. As described in **Methods**, for each topology we determine the specificity, estimated using the hypergeometric distribution, of the most descriptive biological process annotation shared by the proteins in an instance of an emergent schema. For the background set, we enumerate all subgraphs of a given topology in the interaction network, with the restriction that only proteins having at least one Pfam annotation are considered (to avoid bias arising from Pfam annotated proteins). We find that 77% of the instances of the emergent pair schemas share a biological process at the *p*≤0.01 level, as opposed to 53% for the background set. These numbers are 60% vs. 35% for triplet schemas, 87% vs. 69% for triangle schemas, and 58% vs. 21% for Y-star schemas. This enrichment is observed over the entire range of *p*-values (see Figure S1). Functional enrichment is likely due in part to the enrichment of true interactions in emergent schema instances; indeed, interactions from small-scale experiments (<50 interactions uncovered total) are enriched in the emergent pair Pfam schemas instances as compared to the entire interactome.

### Enriched number of *S. cerevisiae* emergent network schemas with instances in *H. sapiens*

In order to determine whether emergent *S. cerevisiae* schemas tend to be found in other organisms, we have used each schema to interrogate the full (i.e., unfiltered) *H. sapiens* physical interaction network in BioGRID [48] and obtain its instances. We limit this analysis to schemas comprised of Pfam annotations that occur in both *S. cerevisiae* and *H. sapiens*. We find that 76% of these *S. cerevisiae* Pfam emergent pair schemas have at least one instance in the *H. sapiens* network. For comparison, if we consider pair schemas with instances in *S. cerevisiae* with FDR>0.05, only 38% have instances in *H. sapiens*. The fraction with instances in *H. sapiens* is 75% for emergent triplet schemas, 61% for emergent triangle schemas, and 55% for emergent Y-star schemas; the instance percentages for schemas not found to be over-represented are 17%, 15%, and 8% respectively. Thus, emergent schemas have instances in *H. sapiens* two to seven times more frequently than schemas of the same topology that are not found to be over-represented, giving further evidence that these schemas correspond to recurring units within interactomes.

### Network schemas in the *H. sapiens* interactome

**Figure 5:**
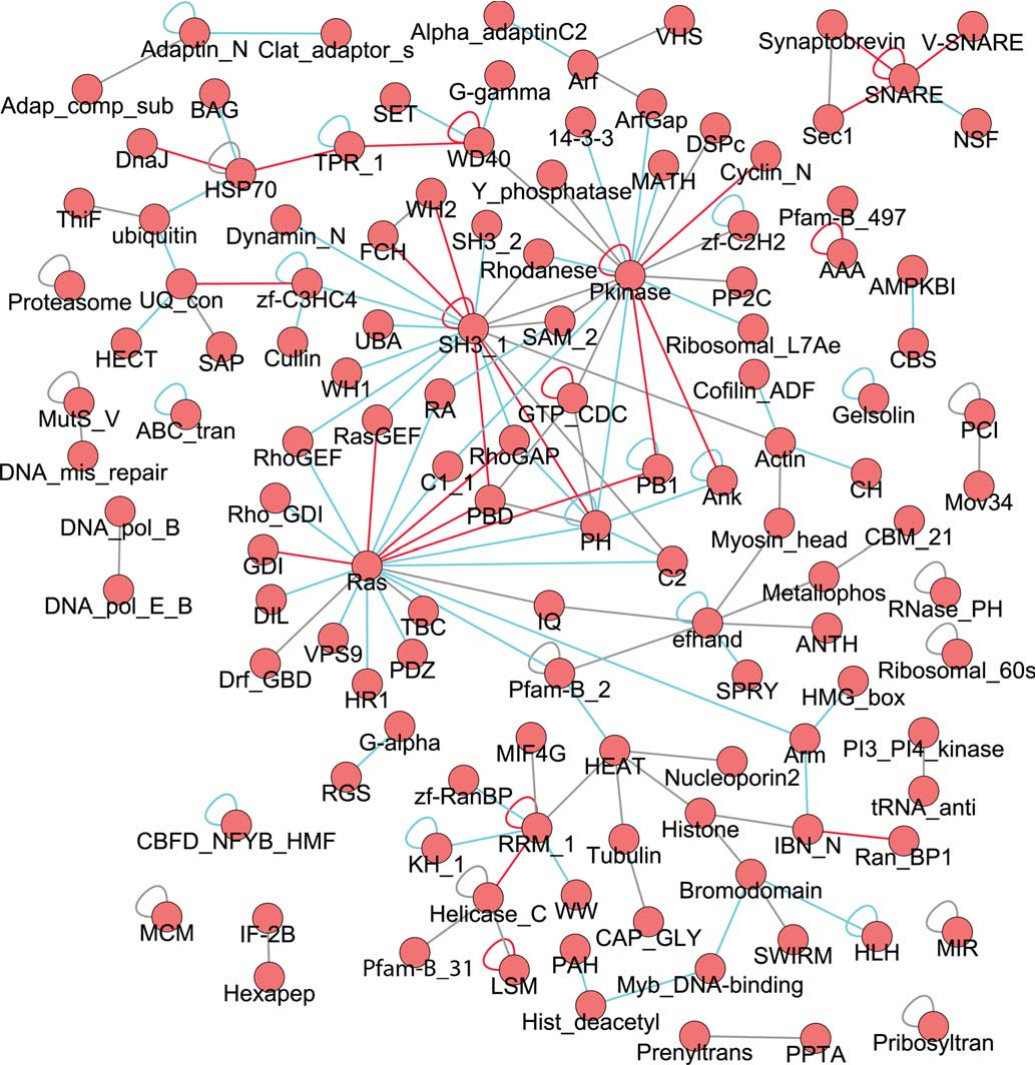
Pfam pair schemas that are found in both *H. sapiens* and *S. cerevisiae*. Schemas that are emergent in both organisms are displayed with red edges. Schemas that are emergent only in *H. sapiens* but that have instances in *S. cerevisiae* are shown with light blue edges. Schemas that are emergent only in *S. cerevisiae* but that have instances in *H. sapiens* are indicated with grey edges.

**Figure 6:**
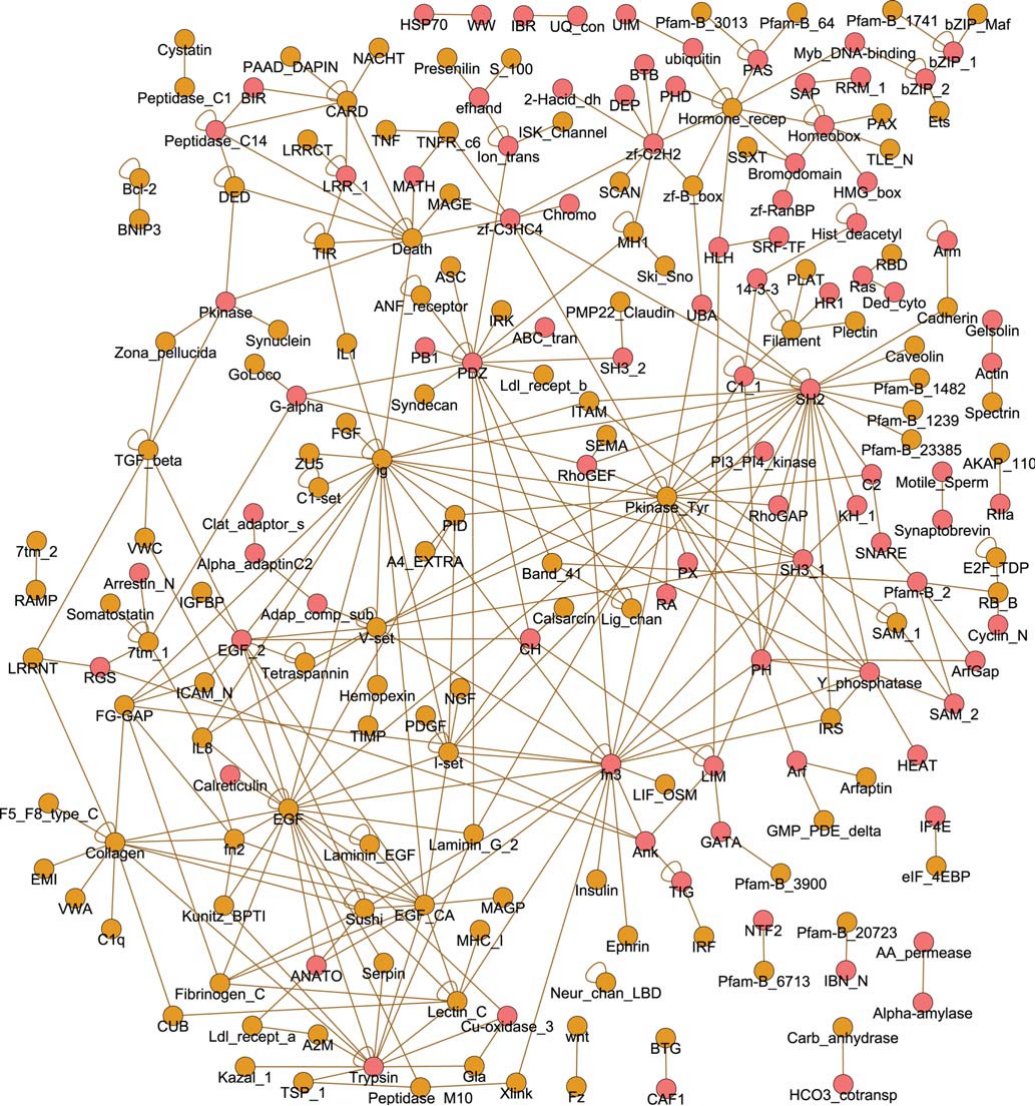
Pfam pair schemas that are emergent in *H. sapiens* and do not have instances in the *S. cerevisiae* interactome. Red vertices indicate Pfam motifs that are found in both organisms, and brown vertices indicate Pfam motifs found in *H. sapiens* but not *S. cerevisiae*.

To compare the types of schemas that are emergent across distant genomes, we uncover pair schemas in the *H. sapiens* interactome (Figures 5 and 6 and Table S7). We identify 29 pair schemas that are emergent schemas in both the *S. cerevisiae* and *H. sapiens* networks, as well as several that are emergent schemas only in *H. sapiens* but have instances in *S. cerevisiae* (Figure 5). As expected, these schemas represent some of the most basic processes that occur within the cell: DNA packaging, cytoskeleton organization, signaling, vesicle fusion, and so on.

The *H. sapiens* emergent pair schemas that are not found in *S. cerevisiae* (Figure 6) contain many schemas related to processes specific to higher organisms. These include, for example, schemas involving the extracellular matrix (e.g., *Collagen* and *Fibrinogen_C* schemas) and intercellular signaling (e.g., *Hormone_recep* schemas), among others. Many of these types of schemas consist of Pfam motifs that are not found in *S. cerevisiae* (e.g., the *Death* domain, found in proteins associated with apoptosis). The *H. sapiens*-specific emergent pair schemas also include some where both motifs are also found in *S. cerevisiae*; some of these schemas correspond to expansions of protein families and their interactions in *H. sapiens*. These include, for example, several emergent schemas involving motifs that are associated with phosphotyrosine signaling (e.g. *SH_2* and *Y_phosphatase* schemas); though these motifs are found in *S. cerevisiae*, they are rare. Additionally, the *H. sapiens* emergent pair schemas reveal how newer motifs, found only in *H. sapiens*, are integrated into networks containing older motifs, found in both organisms. For example, the tyrosine kinase *Pkinase_tyr* motif, found in *H. sapiens* but not *S. cerevisiae*, is involved in emergent pair schemas with signaling domains such as *SH3_1* and *PH* that are found in both organisms.

The *H. sapiens* and *S. cerevisiae* schemas taken together help fill in some of the data missing from the current state of interactomes, as combining the emergent schemas from the two interactomes gives a more complete view for some processes. For example, several schemas relating to ubiquitination consist of pairs that are found to be emergent in only one organism but which have instances in the other; this is most likely due to missing interactions in one of the interactomes. The *S. cerevisiae* emergent schemas cover two parts of the ubiquitination pathway: they include an interaction between the *ubiquitin* family and the *ThiF* family of ubiquitin-activating enzymes, which catalyze the first step of the pathway, and an interaction between the *UQ_con* family of ubiquitin-conjugating enzymes and the *zf-C3HC4* (RING finger) family of ubiquitin ligases, which catalyze the second and third steps of the pathway, respectively. *H. sapiens* emergent schemas that have instances in *S. cerevisiae* complete this portion of the pathway by connecting the ubiquitin family with the *UQ_con* family of ubiquitin-conjugating proteins. Additionally, *H. sapiens* schemas connect *ubiquitin* to the *HSP70* family of chaperones, reflecting the role of ubiquitination in targeting misfolded proteins for degradation.

### Schemas enable functional predictions

There are several motifs of unknown function implicated in schemas (e.g., Pfam-B motifs in Figures 2–5). As proof of concept, we focus on two examples, *DUP* and *MAGE*, and show that schemas can help characterize motifs and proteins whose functions have not yet been experimentally determined.

“One of the most curious gene families in yeast” [49], the *DUP* family consists of twenty-three yeast proteins [50], most of which are not yet functionally annotated. Based on schema analysis, we predict that the *DUP* family consists of proteins that are associated with membrane transporters. The *DUP* proteins are found in multiple schemas of various topologies (Figures 2, 3, and 4), and these schemas are dominated by interactions with members of transporter families such as *MFS_1*, *Sugar_tr*, and *AA_permease*. The finding that one member of the family, Cos3, is an enhancer of the antiporter Nha1p [51] supports this prediction. Additionally, a previous prediction connects *DUP* proteins with membrane trafficking [50]; given our analysis, they might be involved in trafficking of transporters.

**Figure 7:**
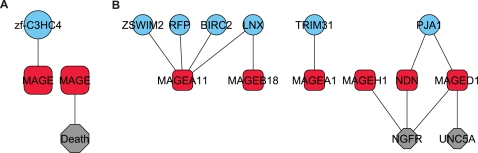
Emergent *H. sapiens* pairs involving the *MAGE* family (A), and their instances (B).

There are fifty-five *MAGE* sequences in *H. sapiens* [52], thirty-two of which are listed as such in Pfam and nine of which have physical interactions listed in BioGRID [48]. *MAGE* proteins, which are mostly uncharacterized, were initially found to be expressed in tumors, although some are now known to be expressed in normal tissues. We found the *MAGE* family to participate in pair schemas with two protein families: the *Death* domain and the *zf-C3HC4* RING motif (see Figures 6 and 7). The *Death* domain is associated with apoptosis, and the RING motif is associated with E3 ubiquitin ligases, which perform the final step in protein ubiquitination. These schemas suggest a connection between *MAGE* proteins and apoptosis, which, if correct, could shed light on the association between some of the original members of the *MAGE* family and cancer. It is possible that ubiquitination plays a role in this connection, although the link between ubiquitination and apoptosis is still a subject of investigation; *MAGE* proteins may provide a connection between these two processes. We further note that the *zf-C3HC4* RING domain forms a schema with the *Death* domain as well (Figure 6).

## Discussion

We have introduced network schemas as a general means to describe organizational units consisting of particular types of proteins that work together in biological networks and have developed a fully-automated procedure for discovering them. In the first analysis of this type, we have uncovered hundreds of emergent network schemas and have demonstrated that they recapitulate known biology, suggest new organizational units, have enriched biological process coherence, and have instances in organisms across large evolutionary distances.

Using two poorly understood gene families, one from human and one from yeast, we have shown how schema analysis can be used to annotate protein families and their individual members. Guilt-by-association and other network-based functional annotation methods (review, [53]) are, by intent and design, better suited for the general function prediction problem. However, schema analysis provides a new way to amplify a weak signal, and can suggest mechanistic details in some cases. For example, if we consider proteins that interact physically with a given protein, and then take the most over-represented biological process annotation among them using the GO Generic Term Finder [54], we find “apoptosis” as a prediction for only two of the *MAGE* proteins having physical interactions (at corrected p-value ≤0.05 level) and no ubiquitination related predictions. On the other hand, schema analysis of the *MAGE* proteins acts as a lens that focuses the investigator's attention on patterns of interaction that together are statistically significant.

The prominence of emergent schemas related to signaling suggests that we may be able to utilize them to uncover pathways. Previous approaches to predicting signaling pathways from protein physical interaction networks have attempted to find paths from receptors to transcription factors [55],[56], and then evaluating them (e.g., based on gene expression coherence [55],[57]). Alternate approaches have attempted to query interactomes in order to find pathways homologous with known pathways [17]. Schemas may instead be used in pathway discovery by restricting or favoring paths in a network based on schema annotations, or using schemas to evaluate or score the enumerated paths. Indeed, simply by taking overlapping emergent network schemas and obtaining their instances in the full unfiltered *S. cerevisiae* interactome, we can recover portions of known pathways. For example, by considering just the triplet schemas *RhoGAP-Ras-RhoGEF*, *RhoGAP-Pkinase-Ras*, and *Ras-Pkinase-SH3_1*, we obtain significant portions of the cell wall organization and biogenesis and cell polarity pathways, and the related pathways of filamentous growth and pheromone response, as well as the cell cycle and vesicle transport pathways (see Figure S2).

Our results can be considered in terms of several alternate hypotheses concerning the evolutionary processes by which schemas arise. Did the different instances of a schema arise from a common ancestral group of interacting proteins which then proliferated, or did convergence play a role? It is likely that both processes took place, with one or the other being more important in different schemas. In the case of Pfam schemas, this question is on the one hand analogous to, and on the other hand intimately related to, the question of how intra-protein domain architectures arose (e.g., see [58],[59]). As a result, the possible role of domain duplications, insertions and shuffling is an important consideration in understanding the evolutionary histories of individual Pfam schemas. For example, in the case of intra-protein domain architectures, graph-theoretic analysis has suggested that combinations involving certain promiscuous domains (SH3 and C2, among others) may have arisen more than once, though other combinations may be the result of the formation of a single ancestral sequence that proliferated through duplication [60]. For schemas that are based on protein annotations that do not necessarily arise from sequence similarity (e.g., GO molecular function schemas), convergence is likely to play a larger role, as the proteins comprising distinct instances may not share any discernable sequence similarity.

Another question that arises is how novel schemas are incorporated over the course of evolution. A comparison of emergent pair schemas in *S. cerevisiae* and *H. sapiens* provides some hints, but further analyses of the interactomes of many organisms is necessary to obtain a better understanding. Similarly, what is the relationship between emergent and non-emergent lower-order schemas that together make up a higher-order emergent schema? Was the non-emergent component added to the earlier emergent one? The techniques introduced in this paper provide a computational foundation for the extensive cross-genomic studies that are necessary to attempt to address these and related questions.

Depending on the intended application it may be desirable to modify the computational procedure for uncovering emergent schemas. The described approach is designed to be conservative in several respects. First, since we search for proteins that work together in a specific topological pattern, we use only networks comprised of direct physical interactions, erring on the side of caution in the case of pull-down data. Alternate approaches may instead be taken to enrich the number of direct interactions but not exclude other types of interactions [61]. Second, we require each emergent schema to have at least two independent instances. Interesting schemas certainly get excluded as a result (e.g., several SCF ubiquitin-ligases in *S. cerevisiae* that differ only in their F-box protein component [62]). Nevertheless, independence helps ensure that an emergent schema is truly recurring and that it does not depend on the occurence of any single interaction; this is an important consideration due to the underlying noise in the network [63]. Finally, we search for emergent schemas bottom-up, eliminating schemas that may owe their significance solely to the significance of their lower-order constituents; this favors including lower-order schemas over higher-order ones. It is possible, however, that in some cases, the higher-order schema is the recurring working unit that makes its lower-order components look significant. Our schema-finding procedure can be modified to relax any of these requirements, and indeed we believe that there are many more functionally important and recurring schemas than we have identified here.

In this work we have examined four of the most basic topologies for schemas. However, additional or flexible topologies (e.g., allowing optional proteins) may also be considered. The primary challenges in extending our current approach lie in computationally enumerating all possible schemas and in developing effective algorithms for maintaining the distribution of the appropriate lower-order constituents. Additionally, whereas here we have considered annotations consisting of Pfam motifs and a subset of GO molecular function terms (each separately), schemas based on several complementary systems of protein labels that annotate at differing levels of resolution may provide a more multidimensional view of protein function; in this case, the hierarchical relationships between annotations would need to be better handled.

A noticeable feature of our analysis is that the underlying data treats all interactions as being the same. In reality, the interactions have both meaning and contextual information. For example, some schemas consist of interactions representing the (de)activation of one of the interactors by the other, with corresponding temporal information. A triplet schema, for example, may correspond to a central protein acting upon its two spoke proteins, or two spoke proteins acting upon the central protein, or one spoke protein acting on the central protein which then acts on the other spoke protein. Schemas may also include a combination of multiple subschemas that are active at different times or in different cellular contexts. Such information is not explicitly present in the schemas we have uncovered and is an especially important consideration when studying multicellular organisms, in which different interactions may take place in different cell types altogether. If contextual information for a large number of interactions becomes known and systematized, it is possible to look for schemas either within each context separately, or include contextual information as part of the schema definition. Alternatively, one could attempt to extract contextual information from the current schemas, focusing on the individual undirected schemas that our approach presently finds, and devising computational means for predicting such information based, for example, on expression information or literature search. Such inclusion of information about the biological context of when interactions occur should refine the network schemas observed. Moving beyond physical interactions, an interesting avenue for future work would be to extend network schemas to specify other types of interaction as well, as has been done for network motifs [12],[13]; the “meaning” or semantics of these types of network schemas would be very different from the type considered here. Schemas uncovered in one type of network can also be used to interrogate other networks. For example, schemas from a physical interaction network may help identify direct interactions in functional networks for organisms for which no large-scale physical interactomes have been determined.

Finally, while here we have searched for emergent schemas in just two sample organisms, our techniques can be applied to a greater number of interactomes across the evolutionary spectrum. This would enable us to uncover what types of schemas are found in different organisms, and to better address how networks expand or change to incorporate new motifs or protein functions. Since large-scale protein interaction networks are being determined at an increasing pace, we anticipate that network schema analysis will become an important means for determining how proteins work together in the cell.

## Methods

### Preliminaries

#### Protein annotations

We use Pfam [14] version 18.0 for motif annotations for all proteins. For *S. cerevisiae* proteins, we additionally consider a set of 134 general molecular function annotations from the Gene Ontology [15]. GO annotations for *S. cerevisiae* proteins are obtained for each sequence from SGD version 1.01 [64] utilizing all evidence codes. These GO terms have been selected by hand to maximize annotation coverage and minimize overlap with respect to GO; see Table S1 for the set of terms.

#### Physical interaction network

We use *S. cerevisiae* and *H. sapiens* protein interaction data from BioGRID [65], release 2.0.20. Since we are interested in uncovering functional units consisting of proteins that work together in specific network topologies, we focus on direct physical interactions by utilizing interactions determined from one of the following experimental systems: Biochemical activity, Co-crystal structure, Far western, FRET, Protein-peptide, Reconstituted complex, and Two-hybrid [64], excluding the IST 1 set of [66]. Additionally, interactions determined via Affinity capture-Western and Affinity capture-MS are used in the case where a bait protein identifies at most one prey. Proteins with ambiguous common names are not used. The physical interaction network is further filtered to remove: (1) interactions from a single experimental source for a protein if that source found over thirty interactions for this protein (2) any proteins with either less than one or more than fifty remaining interactions and (3) any proteins that do not have an annotation that appears at least twice in the remaining interaction network. After all filtering steps, the resulting Pfam-annotated *S. cerevisiae* network has 3,871 interactions among 2,073 proteins described by 472 Pfam terms, and the resulting *H. sapiens* network has 7,284 interactions among 4,062 proteins described by 669 Pfam terms. The same filtering process used with our set of GO molecular function terms on the *S. cerevisiae* interactome leaves 1,834 proteins with 3,542 interactions.

#### Terminology

A protein interaction network is represented as a labeled graph *G* = *V_N_*, *E_N_*), with a vertex *ν*∈*V_N_* for each protein and an edge (*u*, *ν*)∈*E_N_* between vertices whose corresponding proteins interact. Let 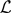 be the set of possible protein annotations (e.g., Pfam motifs). Each protein *v*∈*V_N_* is associated with a set of annotations *l*(*ν*), where 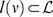. A *network schema* is a graph *H* = (*V_S_*, *E_S_*) where each vertex *ν*∈*V_S_* is specified by a description 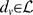. An *instance* of a network schema *H* in an interaction network *G* is a subgraph (*V_I_*, *E_I_*) where *V_I_*⊂*V_N_* and *E_I_*⊂*E_N_* such that there is a one-to-one mapping *f*:*V_S_* → *V_I_* where for each *ν*∈*V_S_*, *d_ν_*∈*l*(*f*(*ν*))and there is an edge (*f*(*u*),*f*(*ν*))∈*E_I_* for each (*u*, *ν*)∈*E_S_* (i.e., it is the match in the network for the schema). Note that two distinct instances of a schema may share proteins and/or interactions; however, any two instances must differ in at least one protein. Two instances of the same network schema are *independent* if they are made up of non-overlapping proteins (i.e., the intersection of their vertex sets is empty). In the case of triplet and Y-star schemas, we allow instances that have additional interactions among the nodes in the interactome (i.e., the endpoints of the triplet or any pair of endpoints of the “spokes” of the Y-star may be connected with an edge). Note that network schemas can be naturally generalized to include other types of interactions and protein annotations [38].

### Uncovering network schemas

The overall procedure for uncovering emergent network schemas of a given topology is as follows; the steps are described in more detail below. First, we count the number of instances of every schema that occurs in the interactome; though this corresponds to the NP-hard subgraph isomorphism problem, we find that in practice we are able to solve it readily [38]. Second, for each schema that has at least two non-overlapping instances, we compute its average number of instances in randomized networks. Third, the schema is scored to favor schemas that both occur frequently and are over-represented compared to their average count in the randomized networks. Fourth, the significance of scores is determined using a false discovery rate that is calculated by repeating the first three steps of the process on randomized networks. Finally, the results are filtered in order to remove redundant schemas.

We developed an extensive algorithmic infrastructure as related techniques are not directly applicable. While there is substantial previous work in the data mining community for frequent (labeled or unlabeled) subgraph mining (e.g., see [67]–[73]), these approaches are focused on the algorithmic issues of enumerating (or eliminating) subgraphs in single or multiple networks, and not on assessing significance or relevance. Here, we are able to take a brute-force approach in enumerating subgraphs, and our methodology development instead is focused on identifying frequent and over-represented subgraphs. We further note that it is not possible simply to apply the approach used for network motif finding [8] to uncover emergent network schemas as well. Specifically, in that approach the count of each network schema in the actual network would be compared to the count in randomized networks, and a *p*-value would be computed by considering what fraction of the randomized networks have a larger number of that network schema; however, this will identify as emergent schemas that occur rarely and are likely to be spurious but are made up of annotations that themselves occur rarely in the network, as these schemas are unlikely to be found in the randomized networks. A similar problem arises with using Z-scores, also reported in [8]. Our scoring and FDR procedure (described below) are designed to better handle the variation in annotation frequency and the large number of schemas of each topology that are considered. Finally, the task of building an ensemble of randomized networks that are constrained to have specified counts of certain labeled subgraphs has not, to the best of our knowledge, been addressed in the past.

#### Randomized networks for computing scores

For each schema *s* that recurs in an interactome (i.e., has at least two instances), we compute how often it occurs in randomized networks, which tells us whether the schema occurs more often than expected by chance. For each pair schema, we count how often it occurs in randomized networks that have been generated using the stub-rewiring approach of [8], which randomizes edges while maintaining the degree and labels of each node in the graph. Note that there is no known efficient method that generates graphs uniformly at random with specified degree and label distribution, so an approximation such as this is used. It is well known that the stub-rewiring procedure may result in networks where some nodes cannot achieve their desired degrees; however, we have found this to be rare in the networks studied here. For example, randomizing the *S. cerevisiae* network 100 times using stub-rewiring, we found that 98 of the random networks had all nodes reaching their original degrees, and 2 of the random networks had two nodes that are below their desired degree by 1. We note that while it is possible to randomize the networks by shuffling annotations while keeping the topology fixed (e.g., as in [28]), annotations have different tendencies to be found in proteins of varying number of interactions, and we wish to maintain this relationship.

For each triplet and triangle schema, we count how often the schema occurs in networks randomized so as to preserve the distributions of the pairs making them up, and for each Y-star schema, we use the same approach, but consider randomized networks that preserve the distribution of triplets making up the Y-star schema (see below). In this manner, we are able to eliminate schemas that are over-represented only because they are comprised of lower-order schemas that are themselves over-represented; instead, we identify schemas that are over-represented even when considering the distribution of the lower-order schemas making them up. As with the stub-rewiring approach, the randomization methods for preserving pair and triplet distributions are approximate, as no efficient algorithms are known for these problems; however, as we show, they work well in practice.

We now describe the subgraph-preserving randomization methods in more detail. For each triplet schema where nodes labeled *a* and *c* interact with a central node labeled *b*, we generate randomized graphs that maintain the original number of interactions between proteins labeled *a* and proteins labeled *b*, and between proteins labeled *b* and proteins labeled *c*. Let these target interaction counts be denoted by *t_ab_* and *t_bc_*, and let *s_ab_* and *s_bc_* be the current count in the network we are generating. The counts of all other pairs of labelings are ignored. To generate the randomized graphs we repeatedly add edges between unconnected proteins, where the probability of adding a particular edge is proportional to how much closer it gets to the desired count of labelings, as measured by the squared L2 distance. That is, if node *u* is labeled with *a* and node *v* is labeled with *b*, an edge between them is added to the graph with probability proportional to max{0,( *t_ab_*−*s_ab_*−*e_uvab_*)^2^}, where *e_uvab_* is the number of *a*−*b* labelings that are introduced by adding an edge between *u* and *v* (in this case *e_uvab_* = 1). Due to the fact that proteins often have multiple annotations, adding an edge may increase the count of more than one of the desired labeling pairs. In this case, the edge is added with probability proportional to the geometric mean of the individual pair labeling scores. We continue adding edges until the pairwise distributions are satisfied or no further edges can be added that can change the number of *a*−*b* or *b*−*c* labellings. As with the stub-rewiring approach, the degree of each protein is maintained, so that an edge is added only if the original degrees of both proteins have not yet been reached. Note that randomized networks are generated separately for each schema, and only edges changing the counts of constituent pair schemas are considered for addition into the network; that is, we only generate a small number of the edges (i.e., those that play a role in the corresponding lower-order schemas). This same process is used to generate randomized graphs for triangle schemas, except that a third pairwise count is also maintained (i.e., the *a*−*c* count). The randomized graphs generated in this manner do an excellent job in achieving the desired distributions. For over 98% of all Pfam triplet schemas that have at least two independent occurrences in the original network (and 96% of triangles), the counts of all their constituent pairs are within one of their counts in the original graph for at least 90% of the randomized graphs.

The same overall scheme is adapted for randomizing networks in order to maintain triplet distributions. In particular, for each Y-star schema where a central node labeled with *a* interacts with nodes labeled with *b*, *c*, and *d*, randomized graphs are generated so as to maintain the number of paths where a protein annotated with *b* interacts with a protein annotated with *a* which in turn interacts with a protein annotated with *c*, the number of paths where a protein annotated with *b* interacts with a protein annotated with *a* which in turn interacts with a protein annotated with *d*, and the number of paths where a protein annotated with *c* interacts with a protein annotated with *a* which in turn interacts with a protein annotated with *d*. We also consider the pairwise interactions in the Y-star; that is, the number of interactions between a protein labeled with *a* with a protein labeled with *b*, as well as the number of interactions with proteins labeled with *c* or *d*. An edge is added with probability proportional to the product of a pair term and a triplet term. As above, degree distributions are maintained and the pairwise (respectively triplet) term for each edge is the geometric mean over each pairwise (respectively triplet) labeling added depending on how much closer that edge gets one to the target count for that labeling. For each possible edge, the triplet term is initialized to be 1 until that edge can contribute to a triplet labeling. Once the pairwise term is 0 for all edges, only the triplet term is considered. This process is continued until the relevant triplet counts for the Y-star are satisfied or until no further edges can be added that can change these counts. At this point, if the randomized network has a triplet that has not reached its target count, we choose a protein that is annotated with the central label with probability proportional to its degree, and choose two proteins with the peripheral labels uniformly at random. New edges are added from the central protein to the two others, removing existing edges if necessary to satisfy the degree distributions. This process is repeated until all triplet target counts are met or exceeded. We find that for over 95% of Pfam Y-star schemas evaluated, the counts of all their constituent triplets are not less than one away from their counts in the original graph for at least 90% of the randomized graphs.

As mentioned, the randomization methods for preserving degree distribution, and pair and/or triplet subschema distributions are approximate and do not come with theoretical guarantees. In order to show that the described randomization procedure produces networks that are sufficiently different from one another (i.e., sample a wide range of possible networks), we take the five top-scoring schemas of triplet, triangle and Y-star topologies (given as the top five entries in Tables S3, S4 and S5) and generate 1000 subschema preserving randomized networks for each of them. We then calculate the overlap between each pair of randomized networks for each schema as the Jacquard coefficient over the edges present in each of the networks. The low average pairwise overlaps (Figure S3) indicate that the randomization procedure is sampling broadly from the set of possible networks. Moreover, we observe that for a given schema the average overlap between subschema preserving networks seems to depend on the number of the target edges desired, the total number of possible edges of the appropriate labels possible, and the degree distribution of the nodes annotated with the labels of interest.

#### Scoring schemas

For each schema *s*, let *count_s_* be the number of times it occurs and *avg_s_* be the average number of times it occurs in randomized networks. The score for schema *s* is given by
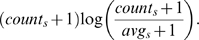
The addition of the pseudocount of 1 downweighs the contribution of very rare schemas that could otherwise obtain high scores simply due to very small (or zero) average counts in the randomized graphs. The scoring function takes into account both a schema's frequency and its over-representation in the real graph compared to the randomized one. While other scoring functions may be utilized, we note that due to the variation in how frequent various annotations are, *count_s_* by itself is not an ideal choice as it favors schemas comprised of frequent annotations.

For each schema, 100 randomized networks are generated, and the average number of times that each schema occurs in these networks is computed. Overall results did not change appreciably when considering more randomizations in this step and keeping the rest of the framework the same (data not shown), suggesting that 100 randomizations are adequate for our purposes. Due to computational concerns, and since we are only interested in independent recurring schemas, scores are computed only for the 419 pair, 842 triplet, 31 triangle, and 999 Y-star schemas that occur independently at least twice in the interactome.

#### Significance model

For each putative recurring schema found in the real network, we obtain a score reflecting its frequency and over-representation compared to the randomized graphs. In order to evaluate the significance of these scores, for each schema topology, we repeat this procedure with multiple *iteration* graphs created by the stub-rewiring algorithm of [8]. Since all associations in these randomized networks occur by chance, we can use them to calculate the FDR for each score, or the fraction of schemas with score ≥*s* that arise from chance alone. For *n* iteration graphs, it can be computed as
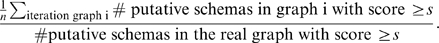
Here, *n* = 50 iteration graphs are used. In order to correct for differences in the clustering coefficient between real and randomized graphs, the FDR of triangle schemas is further corrected by multiplying by the ratio of the number of triangles in the actual network to the average number found in randomized graphs. Note that the false discovery rate corrects for multiple hypothesis testing. We use an FDR of ≤0.05 as the significance cutoff to identify emergent schemas. Note that other FDR values can be used as a cutoff to identify emergent schemas; we choose the 0.05 level as it is a commonly-used one that appears reasonable in this application.

#### Filtering schemas

Once schemas over-represented at FDR≤0.05 are identified, we eliminate any schema for which at least 15% of the randomizations have a labeled subgraph whose count is more than one below its count in the original network. Additionally, the instances for these schemas are obtained and we eliminate those schemas whose instances are a subset of the instances of another schema from the same topology. The remaining schemas are our uncovered emergent schemas.

#### Network alterations

In order to check whether the schemas identified as emergent are robust to changes in the network, we recompute FDRs on the yeast network altered in the following way. First, we remove a percent *x* of the interactions, where each such interaction is chosen uniformly at random. We then add an equal number of interactions, where the two proteins to be connected are again chosen uniformly at random. We consider altered networks with *x* = 2.5%,5.0%,7.5% (i.e., resulting in networks differing from the original network by up to 5%, 10%, 15%, respectively), and generate five altered networks for each of these values. For each perturbed network, the absolute value of the difference in FDRs over all schemas identified as emergent in the original network is computed. Figure S4 gives histograms of these values over all perturbed networks, and shows that the FDRs for most emergent schemas vary very little, with a few outliers. For the networks altered by removing 2.5% and adding 2.5% of the interactions, the median absolute change in FDR over emergent schemas varies from 0.0017 to 0.0036 in the five perturbed networks; these numbers are 0.0018 to 0.0033 when adding and removing 5%, and 0.0019 to 0.0043 when adding and removing 7.5%.

#### Computing requirements

The described schema discovery process is run on a Dell Linux Cluster with 3.2 GHz Xeon and 3.0 GHz Woodcrest processors; 51 total nodes are used (one for the FDR computation of the original network and one for each of the iteration graphs). The entire process for uncovering schemas of the four topologies considered typically takes 12 total hours in a shared user environment.

### Evaluating functional coherence

For each topology, we compile the set of instances of all Pfam emergent schemas. Duplicate instances are removed; for the “background” set, we enumerate all subgraphs of that topology in the same filtered interaction network that is used to search for the Pfam schemas. To avoid any bias that might arise from Pfam annotations, only proteins having at least one Pfam annotation are considered when building the background sets of subgraphs. Furthermore, we require all proteins in each schema instance and each background subgraph to have non-trivial GO biological process annotations; in the case of the Y-star topology, this requirement is relaxed to permit the central node to be unannotated. For each such subgraph, we determine the least common ancestor (LCA) of the annotations of the proteins in the GO biological process graph; if there are multiple LCAs, we select the one that annotates the smallest number of proteins in *S. cerevisiae*. Note that if the proteins are not known to be functionally related, the LCA of their annotations would be the trivial annotation of *biological_process*. The “specificity” of this LCA is calculated as the probability *p* of a schema-sized set of proteins having that annotation, using the hypergeometric distribution. Finally, for a given value of *p*, for both the emergent schema instances and the background set of subgraphs, we can measure the functional coherence of each as the fraction of subgraphs whose constituent proteins have annotations whose LCA specificity is at most *p*.

## Supporting Information

Figure S1 Functional coherence of emergent schema instances compared with arbitrary subgraphs of the same topology. Each panel compares emergent schemas (shown in blue) with a background set of schemas (shown in red) with respect to biological process coherence. As a function of a particular p-value p, we plot the fraction of schema instances that share a biological process term that has p-value less than or equal to p, as judged by the hypergeometric (see text). For all topologies (pairs, first panel; triplets and triangles, second panel; Y-stars, third panel) and over the entire range of p-values, the emergent schemas have a higher fraction of instances with shared biological process than background schemas of the same topology.
(1.48 MB EPS)

Figure S2 An example of using schemas to query pathways. (A) Three overlapping triplet schemas involved in Ras and kinase signaling were chosen. (B) Their instances make up portions of the related pathways of the cell wall organization and biogenesis, cell polarity, filamentous growth, pheromone response, cell cycle, and vesicle transport pathways. Shapes represent Pfam motifs, and colored circles correspond to GO biological process annotations. The figure is drawn using Cytoscape [74] with the GOlorize plugin [75] for functional coloring.
(0.29 MB EPS)

Figure S3 Variation in 1000 subschema preserving randomized networks for each of the five top-scoring triplet, triangle, and Y-star schemas. Each point in the graph plots the average Jacquard coefficient between pairs of networks randomized for the same schema, with error bars showing plus and minus one standard deviation, as a function of the total target number of edges desired between proteins of particular annotations divided by the total number of possible edges having those annotations. The overlap between randomized networks also appears to depend on the degree distribution of the proteins with the relevant labels (not depicted here).
(0.24 MB EPS)

Figure S4 A histogram of the absolute differences between the FDRs of emergent schemas in the original network and a network altered by removing and then adding 2.5% (top), 5.0% (middle) and 7.5% (bottom) of the edges. Results in each histogram are aggregated over five altered networks, and the heights of the bars give the number of schemas falling into the five bins corresponding to changes in FDR<0.1, 0.03, 0.05, 0.1 and 1.0.
(0.26 MB EPS)

Table S1 GO molecular function terms used
(0.02 MB PDF)

Table S2 Emergent S. cerevisiae Pfam pair schemas
(0.03 MB PDF)

Table S3 Emergent S. cerevisiae Pfam triplet schemas
(0.02 MB PDF)

Table S4 Emergent S. cerevisiae Pfam triangle schemas
(0.01 MB PDF)

Table S5 Emergent S. cerevisiae Pfam Y-star schemas
(0.02 MB PDF)

Table S6 Emergent S. cerevisiae GO molecular function pair schemas
(0.02 MB PDF)

Table S7 Emergent H. sapiens Pfam pair schemas
(0.05 MB PDF)

Table S8 Table S8a (top) gives the most frequent Pfam motifs in the filtered yeast interactome, along with the number of proteins they annotate. Table S8b (bottom) gives the Pfam motifs that take part in the most number of interactions in the filtered yeast interactome, computed as the sum of the degrees of all proteins annotated with the terms.
(0.01 MB PDF)

Table S9 Instances of emergent pfam pair schemas in S cerevisiae
(0.03 MB TXT)

Table S10 Instances of emergent pfam triplet schemas in S cerevisiae
(0.05 MB TXT)

Table S11 Instances of emergent Pfam triangle schemas in S cerevisiae
(0.01 MB TXT)

Table S12 Instances of emergent pfam Y-star schemas in S cerevisiae
(0.04 MB TXT)
