# Supplementary material for: Organization of Physical Interactomes as Uncovered by Network Schemas
Source: PLoS Comput Biol. 2008 Oct 24;4(10):e1000203. doi: 10.1371/journal.pcbi.1000203 (PMC2561054; doi:10.1371/journal.pcbi.1000203)
Supplement: Table S1 — GO molecular function terms used (0.02 MB PDF) [file pcbi.1000203.s005.pdf]

ID	name
GO:0003743	translation initiation factor activity
GO:0003689	DNA clamp loader activity
GO:0003746	translation elongation factor activity
GO:0043021	ribonucleoprotein binding
GO:0003755	peptidyl-prolyl cis-trans isomerase activity
GO:0003756	protein disulfide isomerase activity
GO:0015297	antiporter activity
GO:0031386	protein tag
GO:0003777	microtubule motor activity
GO:0003779	actin binding
GO:0043130	ubiquitin binding
GO:0042626	ATPase activity coupled to transmembrane movement of substances
GO:0003916	DNA topoisomerase activity
GO:0003873	6-phosphofructo-2-kinase activity
GO:0003923	GPI-anchor transamidase activity
GO:0003924	GTPase activity
GO:0004428	inositol or phosphatidylinositol kinase activity
GO:0003887	DNA-directed DNA polymerase activity
GO:0009982	pseudouridine synthase activity
GO:0004396	hexokinase activity
GO:0003899	DNA-directed RNA polymerase activity
GO:0003964	RNA-directed DNA polymerase activity
GO:0004467	long-chain-fatty-acid-CoA ligase activity
GO:0042736	NADH kinase activity
GO:0004519	endonuclease activity
GO:0004527	exonuclease activity
GO:0004540	ribonuclease activity
GO:0004536	deoxyribonuclease activity
GO:0015631	tubulin binding
GO:0004497	monooxygenase activity
GO:0004553	hydrolase activity hydrolyzing O-glycosyl compounds
GO:0005048	signal sequence binding
GO:0005057	receptor signaling protein activity
GO:0016209	antioxidant activity
GO:0005085	guanyl-nucleotide exchange factor activity
GO:0005092	GDP-dissociation inhibitor activity
GO:0005095	GTPase inhibitor activity
GO:0005200	structural constituent of cytoskeleton
GO:0005096	GTPase activator activity
GO:0004652	polynucleotide adenylyltransferase activity

GO:0004721	phosphoprotein phosphatase activity
GO:0004672	protein kinase activity
GO:0004739	pyruvate dehydrogenase (acetyl-transferring) activity
GO:0004748	ribonucleoside-diphosphate reductase activity
GO:0004749	ribose phosphate diphosphokinase activity
GO:0005199	structural constituent of cell wall
GO:0004812	aminoacyl-tRNA ligase activity
GO:0005319	lipid transporter activity
GO:0005275	amine transporter activity
GO:0005342	organic acid transporter activity
GO:0015932	nucleobase nucleoside nucleotide and nucleic acid transporter activity
GO:0043565	sequence-specific DNA binding
GO:0043566	structure-specific DNA binding
GO:0005372	water transporter activity
GO:0004872	receptor activity
GO:0017022	myosin binding
GO:0005507	copper ion binding
GO:0005509	calcium ion binding
GO:0017056	structural constituent of nuclear pore
GO:0005524	ATP binding
GO:0005519	cytoskeletal regulatory protein binding
GO:0005525	GTP binding
GO:0016564	transcriptional repressor activity
GO:0005485	v-SNARE activity
GO:0017069	snRNA binding
GO:0005486	t-SNARE activity
GO:0016702	oxidoreductase activity acting on single donors with incorporation of molecular oxygen incor
GO:0016722	oxidoreductase activity oxidizing metal ions
GO:0016667	oxidoreductase activity acting on sulfur group of donors
GO:0000049	tRNA binding
GO:0016746	transferase activity transferring acyl groups
GO:0016757	transferase activity transferring glycosyl groups
GO:0016765	transferase activity transferring alkyl or aryl (other than methyl) groups
GO:0000150	recombinase activity
GO:0000146	microfilament motor activity
GO:0016830	carbon-carbon lyase activity
GO:0000149	SNARE binding
GO:0016790	thiolester hydrolase activity
GO:0016835	carbon-oxygen lyase activity
GO:0016789	carboxylic ester hydrolase activity
GO:0016868	intramolecular transferase activity phosphotransferases

GO:0000339	RNA cap binding
GO:0051020	GTPase binding
GO:0051082	unfolded protein binding
GO:0051087	chaperone binding
GO:0035091	phosphoinositide binding
GO:0051183	vitamin transporter activity
GO:0051184	cofactor transporter activity
GO:0019207	kinase regulator activity
GO:0019208	phosphatase regulator activity
GO:0019213	deacetylase activity
GO:0008081	phosphoric diester hydrolase activity
GO:0008134	transcription factor binding
GO:0008079	translation termination factor activity
GO:0008168	methyltransferase activity
GO:0008177	succinate dehydrogenase (ubiquinone) activity
GO:0008233	peptidase activity
GO:0008301	DNA bending activity
GO:0001671	ATPase stimulator activity
GO:0008270	zinc ion binding
GO:0008324	cation transporter activity
GO:0008509	anion transporter activity
GO:0008483	transaminase activity
GO:0009055	electron carrier activity
GO:0008565	protein transporter activity
GO:0008639	small protein conjugating enzyme activity
GO:0030188	chaperone regulator activity
GO:0046873	metal ion transporter activity
GO:0047429	nucleoside-triphosphate diphosphatase activity
GO:0030276	clathrin binding
GO:0019787	ubiquitin-like-protein ligase activity
GO:0019843	rRNA binding
GO:0030371	translation repressor activity
GO:0030515	snoRNA binding
GO:0042124	1 3-beta-glucanosyltransferase activity
GO:0015036	disulfide oxidoreductase activity
GO:0031202	RNA splicing factor activity transesterification mechanism
GO:0030674	protein binding bridging
GO:0015144	carbohydrate transporter activity
GO:0004088	carbamoyl-phosphate synthase (glutamine-hydrolyzing) activity
GO:0003700	transcription factor activity
GO:0003701	RNA polymerase I transcription factor activity

GO:0003702	RNA polymerase II transcription factor activity
GO:0015238	drug transporter activity
GO:0003711	transcriptional elongation regulator activity
GO:0015197	peptide transporter activity
GO:0003709	RNA polymerase III transcription factor activity
GO:0003724	RNA helicase activity
GO:0042393	histone binding
GO:0003682	chromatin binding
GO:0003678	DNA helicase activity
GO:0003684	damaged DNA binding
GO:0003729	mRNA binding
GO:0003735	structural constituent of ribosome
