# Supplementary material for: Organization of Physical Interactomes as Uncovered by Network Schemas
Source: PLoS Comput Biol. 2008 Oct 24;4(10):e1000203. doi: 10.1371/journal.pcbi.1000203 (PMC2561054; doi:10.1371/journal.pcbi.1000203)
Supplement: Table S2 — Emergent S. cerevisiae Pfam pair schemas (0.03 MB PDF) [file pcbi.1000203.s006.pdf]

ID_1	Name_1	ID_2	Name_2	FDR	Num in yeast	Avg num in random networks	Instance in human?
PB000002	Pfam-B_2	PB000002	Pfam-B_2	0	7	0.58	Y
PB000002	Pfam-B_2	PF02463	SMC_N	0	8	0.42	N
PB000497	Pfam-B_497	PF00004	AAA	0	6	0.13	Y
PB000521	Pfam-B_521	PF01105	EMP24_GP25L	0	6	0.47	N
PB006792	PB006792	PF00069	Pkinase	0	7	1.01	N
PB042766	PB042766	PF00069	Pkinase	0	10	1.15	N
PF00004	AAA	PF00004	AAA	0	13	0.67	Y
PF00012	HSP70	PF00226	DnaJ	0	6	0.15	Y
PF00018	SH3_1	PF00018	SH3_1	0	24	5.51	Y
PF00018	SH3_1	PF00069	Pkinase	0	24	15.55	Y
PF00018	SH3_1	PF00168	C2	0	8	1.66	Y
PF00018	SH3_1	PF00169	PH	0	10	2.79	Y
PF00018	SH3_1	PF00786	PBD	0	9	2.28	Y
PF00018	SH3_1	PF02205	WH2	0	17	1.26	Y
PF00022	Actin	PF00063	Myosin_head	0	5	0.23	Y
PF00025	Arf	PF01412	ArfGap	0	5	0.04	Y
PF00036	efhand	PF00063	Myosin_head	0	6	0.54	Y
PF00036	efhand	PF00612	IQ	0	5	0.24	Y
PF00069	Pkinase	PF00069	Pkinase	0	49	14.66	Y
PF00069	Pkinase	PF00096	zf-C2H2	0	15	4.16	Y
PF00069	Pkinase	PF00134	Cyclin_N	0	15	3.28	Y
PF00069	Pkinase	PF00735	GTP_CDC	0	13	3.44	Y
PF00069	Pkinase	PF00782	DSPc	0	7	0.97	Y
PF00069	Pkinase	PF03637	Mob1_phocein	0	6	0.46	N
PF00071	Ras	PF00412	LIM	0	9	0.33	N
PF00071	Ras	PF00566	TBC	0	14	0.83	Y
PF00071	Ras	PF00617	RasGEF	0	7	0.47	Y
PF00071	Ras	PF00620	RhoGAP	0	17	0.72	Y
PF00071	Ras	PF00996	GDI	0	12	0.44	Y
PF00071	Ras	PF04893	Yip1	0	17	0.91	N
PF00076	RRM_1	PF00076	RRM_1	0	9	1.36	Y
PF00076	RRM_1	PF00271	Helicase_C	0	8	2.21	Y
PF00083	Sugar_tr	PF00674	PF00674	0	10	1.7	N
PF00097	zf-C3HC4	PF00179	UQ_con	0	8	0.16	Y
PF00125	Histone	PF00125	Histone	0	6	0.32	N
PF00125	Histone	PF00439	Bromodomain	0	12	0.28	Y
PF00125	Histone	PF00583	Acetyltransf_1	0	8	0.21	N
PF00125	Histone	PF02985	HEAT	0	6	0.77	Y
PF00125	Histone	PF03810	IBN_N	0	6	0.46	Y
PF00169	PH	PB063688	PB063688	0	5	0.29	N
PF00169	PH	PF00786	PBD	0	6	0.47	Y
PF00172	PF00172	PF00172	PF00172	0	7	0.35	N
PF00227	Proteasome	PF00227	Proteasome	0	14	0.1	Y
PF00320	GATA	PF00454	PI3_PI4_kinase	0	5	0.12	N
PF00324	AA_permease	PF00674	PF00674	0	14	1.91	N
PF00324	AA_permease	PF01105	EMP24_GP25L	0	10	1.01	N

PF00324	AA_permease	PF03198	PF03198	0	7	0.77	N
PF00400	WD40	PF00400	WD40	0	8	2.05	Y
PF00674	PF00674	PF00674	PF00674	0	7	1.26	N
PF00735	GTP_CDC	PF00735	GTP_CDC	0	9	0.15	Y
PF00735	GTP_CDC	PF00786	PBD	0	8	0.47	Y
PF00786	PBD	PB063688	PB063688	0	5	0.25	N
PF00957	Synaptobrevin	PF05008	V-SNARE	0	7	0.26	N
PF01174	PF01174	PF01680	PF01680	0	7	0.02	N
PF01398	Mov34	PF01399	PCI	0	5	0.09	Y
PF01423	LSM	PF01423	LSM	0	41	2.75	Y
PF02463	SMC_N	PF02463	SMC_N	0	6	0.07	N
PF02985	HEAT	PF04096	Nucleoporin2	0	6	0.23	Y
PF05739	SNARE	PF00957	Synaptobrevin	0	18	0.78	Y
PF05739	SNARE	PF00995	Sec1	0	7	0.22	Y
PF05739	SNARE	PF05008	V-SNARE	0	11	0.4	Y
PF05739	SNARE	PF05739	SNARE	0	6	0.56	Y
PF06371	Drf_GBD	PF00018	SH3_1	0	8	0.91	N
PF07647	SAM_2	PF00069	Pkinase	0	9	1.84	Y
PF07690	MFS_1	PF00137	ATP-synt_C	0	9	0.52	N
PF07690	MFS_1	PF00674	PF00674	0	20	2.41	N
PF07690	MFS_1	PF01105	EMP24_GP25L	0	8	1.32	N
PB046617	PB046617	PF00957	Synaptobrevin	0.01	4	0.14	N
PB073262	PB073262	PF00169	PH	0.01	4	0.15	N
PF00012	HSP70	PF00515	TPR_1	0.01	5	0.62	Y
PF00018	SH3_1	PF00611	FCH	0.01	7	1.61	Y
PF00025	Arf	PF00790	VHS	0.01	4	0.07	Y
PF00069	Pkinase	PF00400	WD40	0.01	17	10.91	Y
PF00071	Ras	PF00612	IQ	0.01	5	0.46	Y
PF00071	Ras	PF00786	PBD	0.01	6	1.25	Y
PF00076	RRM_1	PF02854	MIF4G	0.01	5	0.34	Y
PF00091	Tubulin	PF01302	CAP_GLY	0.01	4	0.02	Y
PF00096	zf-C2H2	PF00515	TPR_1	0.01	5	0.52	N
PF00102	Y_phosphatase	PF00069	Pkinase	0.01	5	0.32	Y
PF00136	DNA_pol_B	PF04042	DNA_pol_E_B	0.01	4	0	Y
PF00153	Mito_carr	PF00674	PF00674	0	6	0.76	N
PF00149	Metallophos	PF03095	PTPA	0.01	4	0.06	N
PF00156	Pribosyltran	PF00156	Pribosyltran	0.01	4	0.02	Y
PF00169	PH	PF00735	GTP_CDC	0.01	5	0.48	Y
PF00179	UQ_con	PF02037	SAP	0.01	4	0.06	Y
PF00271	Helicase_C	PF00271	Helicase_C	0.01	5	0.62	N
PF00271	Helicase_C	PF01423	LSM	0.01	8	2.66	Y
PF00400	WD40	PF00515	TPR_1	0.01	7	1.83	Y
PF00439	Bromodomain	PF04433	SWIRM	0.01	4	0.02	Y
PF00454	PI3_PI4_kinase	PF00498	FHA	0.01	4	0.06	N
PF00454	PI3_PI4_kinase	PF01336	tRNA_anti	0.01	4	0.07	Y
PF00493	MCM	PF00493	MCM	0.01	4	0.03	Y
PF00564	PB1	PF00786	PBD	0.01	4	0.11	N
PF00928	Adap_comp_sub	PF01602	Adaptin_N	0.01	4	0.02	Y

PF00957	Synaptobrevin	PF00995	Sec1	0.01	4	0.11	Y
PF01105	EMP24_GP25L	PF01066	CDP-OH_P_transf	0.01	5	0.4	N
PF03162	PF03162	PF03162	PF03162	0.01	4	0	N
PF06398	PF06398	PF06398	PF06398	0.01	4	0	N
PB000002	Pfam-B_2	PF00036	efhand	0.02	5	0.96	Y
PB000031	Pfam-B_31	PF00271	Helicase_C	0.02	4	0.25	Y
PB000521	Pfam-B_521	PF04811	Sec23_trunk	0.02	4	0.28	N
PB028889	PB028889	PF00069	Pkinase	0.02	4	0.32	N
PF00005	ABC_tran	PF01423	LSM	0.02	6	1.65	N
PF00018	SH3_1	PF00581	Rhodanese	0.02	5	0.79	Y
PF00022	Actin	PF00018	SH3_1	0.02	6	1.6	Y
PF00023	Ank	PF00069	Pkinase	0.02	7	2.26	Y
PF00069	Pkinase	PF00481	PP2C	0.02	5	0.87	Y
PF00069	Pkinase	PF04153	NOT2_3_5	0.02	5	0.91	N
PF00071	Ras	PF00564	PB1	0.02	4	0.37	Y
PF00076	RRM_1	PF02985	HEAT	0.02	6	1.63	Y
PF00083	Sugar_tr	PF00515	TPR_1	0.02	5	1	N
PF00091	Tubulin	PF02985	HEAT	0.02	4	0.17	Y
PF00611	FCH	PF02205	WH2	0.02	4	0.2	Y
PF00787	PX	PF04893	Yip1	0.02	4	0.25	N
PF07651	ANTH	PF00036	efhand	0.02	4	0.18	Y
PF08171	PF08171	PF00400	WD40	0.02	4	0.2	N
PF00069	Pkinase	PF00224	PK	0.03	4	0.42	N
PF00149	Metallophos	PF00439	Bromodomain	0.03	4	0.47	N
PF00170	bZIP_1	PF02985	HEAT	0.03	4	0.43	N
PF01105	EMP24_GP25L	PF01598	Sterol_desat	0.03	4	0.39	N
PF07647	SAM_2	PF00018	SH3_1	0.03	5	1.06	Y
PF07690	MFS_1	PF01598	Sterol_desat	0.03	4	0.45	N
PF00009	GTP_EFTU	PF01423	LSM	0.04	5	1.28	N
PF00069	Pkinase	PF00564	PB1	0.04	5	1.28	Y
PF06371	Drf_GBD	PF00071	Ras	0.04	4	0.58	Y
PB008540	PB008540	PF02133	PF02133	0.05	3	0.04	N
PF00012	HSP70	PF00012	HSP70	0.05	3	0.06	Y
PF00036	efhand	PF00149	Metallophos	0.05	4	0.69	Y
PF00085	Thioredoxin	PF00578	AhpC-TSA	0.05	3	0.02	N
PF00132	Hexapep	PF01008	IF-2B	0.05	3	0.01	Y
PF00149	Metallophos	PF03370	CBM_21	0.05	3	0.07	Y
PF00240	ubiquitin	PF00899	ThiF	0.05	3	0.04	Y
PF00428	Ribosomal_60s	PF00428	Ribosomal_60s	0.05	3	0	Y
PF00432	Prenyltrans	PF01239	PPTA	0.05	3	0	Y
PF00488	MutS_V	PF00488	MutS_V	0.05	3	0.03	Y
PF00488	MutS_V	PF01119	DNA_mis_repair	0.05	3	0.02	Y
PF00562	RNA_pol_Rpb2_6	PF01096	TFIIS_C	0.05	3	0.01	N
PF00562	RNA_pol_Rpb2_6	PF01193	RNA_pol_L	0.05	3	0.03	N
PF00638	Ran_BP1	PF03810	IBN_N	0.05	3	0.06	Y
PF01066	CDP-OH_P_transf	PF00674	PF00674	0.05	4	0.65	N
PF01138	RNase_PH	PF01138	RNase_PH	0.05	3	0	Y
PF01193	RNA_pol_L	PF01193	RNA_pol_L	0.05	3	0	N

PF01399	PCI	PF01399	PCI	0.05	3	0.03	Y
PF01853	MOZ_SAS	PF03366	YEATS	0.05	3	0	N
PF01920	KE2	PF02996	Prefoldin	0.05	3	0	N
PF02466	Tim17	PF02953	zf-Tim10_DDP	0.05	3	0	N
PF02815	MIR	PF02815	MIR	0.05	3	0.01	Y
PF03357	ESCRT-III	PF03357	ESCRT-III	0.05	3	0	N
PF03911	Sec61_beta	PF04756	OST3_OST6	0.05	3	0.03	N
PF07651	ANTH	PF08226	PF08226	0.05	3	0.04	N
PF08240	ADH_N	PF08240	ADH_N	0.05	3	0.02	N
