# Supplementary material for: Organization of Physical Interactomes as Uncovered by Network Schemas
Source: PLoS Comput Biol. 2008 Oct 24;4(10):e1000203. doi: 10.1371/journal.pcbi.1000203 (PMC2561054; doi:10.1371/journal.pcbi.1000203)
Supplement: Table S3 — Emergent S. cerevisiae Pfam triplet schemas (0.02 MB PDF) [file pcbi.1000203.s007.pdf]

ID_1	Name_1	Center ID_2	Name_2	ID_3	Name_3	FDR	Count in yeast	Avg count in random	Instance in human?
PB006792	PB006792	PF00069	Pkinase	PF00069	Pkinase	0	33	6.39	N
PB006792	PB006792	PF00069	Pkinase	PF00134	Cyclin_N	0	36	1.05	N
PF00069	Pkinase	PF00069	Pkinase	PF00134	Cyclin_N	0	57	13.86	Y
PF01423	LSM	PF01423	LSM	PF01423	LSM	0	254	206.3	Y
PF07690	MFS_1	PF00674	PF00674	PF07690	MFS_1	0.01	43	8.47	N
PF00018	SH3_1	PF00069	Pkinase	PF00069	Pkinase	0	59	25.38	Y
PF00069	Pkinase	PF00069	Pkinase	PF00069	Pkinase	0	73	42.86	Y
PF00069	Pkinase	PF00071	Ras	PF00620	RhoGAP	0	24	4.7	Y
PF00096	zf-C2H2	PF00069	Pkinase	PF00096	zf-C2H2	0	16	0.71	Y
PF00125	Histone	PF00439	Bromodomain	PF00125	Histone	0	25	5.24	N
PF07647	SAM_2	PF00069	Pkinase	PF00018	SH3_1	0	22	1.98	Y
PF07690	MFS_1	PF00674	PF00674	PF00324	AA_permease	0	44	15.69	N
PF07690	MFS_1	PF00674	PF00674	PF00674	PF00674	0	43	15.55	N
PB042766	PB042766	PF00069	Pkinase	PF00069	Pkinase	0.01	28	9.1	N
PF00018	SH3_1	PF00018	SH3_1	PF00069	Pkinase	0.01	82	55.64	Y
PF00018	SH3_1	PF00018	SH3_1	PF02205	WH2	0.01	59	35.54	Y
PF00018	SH3_1	PF00069	Pkinase	PF00018	SH3_1	0.01	21	3.21	Y
PF00018	SH3_1	PF00069	Pkinase	PF00169	PH	0.01	14	1.67	Y
PF00018	SH3_1	PF00069	Pkinase	PF00564	PB1	0.01	13	1.12	Y
PF00018	SH3_1	PF00071	Ras	PF00620	RhoGAP	0.01	20	4.61	Y
PF00069	Pkinase	PF00439	Bromodomain	PF00125	Histone	0.01	16	3.07	N
PF00071	Ras	PF04893	Yip1	PF00071	Ras	0.01	64	43.36	N
PF00096	zf-C2H2	PF00069	Pkinase	PF00172	PF00172	0.01	14	0.65	N
PF00134	Cyclin_N	PF00069	Pkinase	PF00096	zf-C2H2	0.01	14	1.88	Y
PF00137	ATP-synt_C	PF07690	MFS_1	PF00674	PF00674	0.01	19	4.57	N
PF00271	Helicase_C	PF01423	LSM	PF01423	LSM	0.01	61	40.18	Y
PF00564	PB1	PF00071	Ras	PF00620	RhoGAP	0.01	16	2.52	Y
PF00620	RhoGAP	PF00071	Ras	PF00620	RhoGAP	0.01	23	4.74	Y
PF00735	GTP_CDC	PF00069	Pkinase	PF00735	GTP_CDC	0.01	11	0.58	N
PF00069	Pkinase	PF00069	Pkinase	PF00096	zf-C2H2	0.02	31	14.43	Y
PF00018	SH3_1	PF00018	SH3_1	PF00018	SH3_1	0.03	63	44.99	Y
PF00137	ATP-synt_C	PF07690	MFS_1	PF01598	Sterol_desat	0.03	10	0.76	N
PF00620	RhoGAP	PF00071	Ras	PF00621	RhoGEF	0.03	12	2.01	Y
PF00620	RhoGAP	PF00071	Ras	PF00787	PX	0.03	13	2.46	Y
PF07690	MFS_1	PB000521	Pfam-B_521	PF00324	AA_permease	0.03	28	13.58	N
PF00018	SH3_1	PF00069	Pkinase	PF00621	RhoGEF	0.04	10	0.95	Y
PF00022	Actin	PF00018	SH3_1	PF00018	SH3_1	0.04	26	12.34	Y
PF00069	Pkinase	PF00069	Pkinase	PF00786	PBD	0.04	16	5.09	Y
PF00069	Pkinase	PF00071	Ras	PF00412	LIM	0.04	12	2.55	N
PF00076	RRM_1	PF01423	LSM	PF01423	LSM	0.04	39	25.01	Y
PF00134	Cyclin_N	PF00069	Pkinase	PF00320	GATA	0.04	8	0.22	N
PF00137	ATP-synt_C	PF07690	MFS_1	PF01105	EMP24_GP25L	0.04	11	1.86	N
PF00169	PH	PF00069	Pkinase	PF00564	PB1	0.04	8	0.31	Y
PF00674	PF00674	PF07690	MFS_1	PF01598	Sterol_desat	0.04	11	1.8	N
PF01105	EMP24_GP25L	PF07690	MFS_1	PF00674	PF00674	0.04	15	4.01	N
PF06371	Drf_GBD	PF00018	SH3_1	PF00018	SH3_1	0.04	31	17.18	N
PF07647	SAM_2	PF00069	Pkinase	PF00564	PB1	0.04	8	0.37	Y
PF00018	SH3_1	PF00069	Pkinase	PF00071	Ras	0.05	11	1.71	Y
PF00018	SH3_1	PF00069	Pkinase	PF00400	WD40	0.05	14	3.93	Y
PF00018	SH3_1	PF00069	Pkinase	PF00735	GTP_CDC	0.05	13	3.43	N
PF00069	Pkinase	PF00069	Pkinase	PF00169	PH	0.05	18	7.3	Y
PF00125	Histone	PF02985	HEAT	PF00170	bZIP_1	0.05	9	1.08	N
PF00134	Cyclin_N	PF00069	Pkinase	PF00621	RhoGEF	0.05	8	0.45	N
PF00400	WD40	PF01423	LSM	PF01423	LSM	0.05	43	29.54	Y
PF00996	GDI	PF00071	Ras	PF04893	Yip1	0.05	19	7.69	N
