# Supplementary material for: Organization of Physical Interactomes as Uncovered by Network Schemas
Source: PLoS Comput Biol. 2008 Oct 24;4(10):e1000203. doi: 10.1371/journal.pcbi.1000203 (PMC2561054; doi:10.1371/journal.pcbi.1000203)
Supplement: Table S4 — Emergent S. cerevisiae Pfam triangle schemas (0.01 MB PDF) [file pcbi.1000203.s008.pdf]

**ID_1**	**Name_1**	**ID_2**	**Name_2**	**ID_3**	**Name_3**	**FDR**	**Count in yeast**	**Avg count in random**	**Instance in human?**
PF00018	SH3_1	PF00018	SH3_1	PF02205	WH2	0	22	5.54	Y
PF00069	Pkinase	PF00735	GTP_CDC	PF00735	GTP_CDC	0	9	0.22	N
PF00125	Histone	PF00125	Histone	PF00439	Bromodomain	0	10	0.75	N
PF01423	LSM	PF01423	LSM	PF01423	LSM	0	64	26.65	Y
PF05739	SNARE	PF00957	Synaptobrevin	PF05008	V-SNARE	0	17	3.73	N
PB000521	Pfam-B_521	PF00324	AA_permease	PF00674	PF00674	0	4	0.04	N
PB042766	PB042766	PF00069	Pkinase	PF00069	Pkinase	0	3	0.13	N
PF00004	AAA	PF00004	AAA	PF00004	AAA	0	4	0.03	Y
PF00018	SH3_1	PF00018	SH3_1	PF00018	SH3_1	0	7	1.24	Y
PF00018	SH3_1	PF00018	SH3_1	PF00069	Pkinase	0	4	0.16	Y
PF00018	SH3_1	PF00018	SH3_1	PF00071	Ras	0	3	0.05	Y
PF00022	Actin	PF00018	SH3_1	PF00018	SH3_1	0	3	0.07	N
PF00069	Pkinase	PF00069	Pkinase	PF00069	Pkinase	0	3	0.1	Y
PF00069	Pkinase	PF00069	Pkinase	PF00134	Cyclin_N	0	5	0.03	Y
PF00069	Pkinase	PF00169	PH	PF00564	PB1	0	3	0	Y
PF00169	PH	PF00564	PB1	PF00786	PBD	0	3	0.31	N
PF00324	AA_permease	PF01105	EMP24_GP25L	PF01598	Sterol_desat	0	3	0.26	N
PF00735	GTP_CDC	PF00735	GTP_CDC	PF00786	PBD	0	7	1.63	N
PF05739	SNARE	PF00957	Synaptobrevin	PF00995	Sec1	0	7	0.64	Y
PF05739	SNARE	PF05739	SNARE	PF00957	Synaptobrevin	0	7	2.54	Y
PF05739	SNARE	PF05739	SNARE	PF05008	V-SNARE	0	7	2.07	Y
PF07647	SAM_2	PF00018	SH3_1	PF00069	Pkinase	0	3	0.02	Y
PF07690	MFS_1	PF00137	ATP-synt_C	PF01105	EMP24_GP25L	0	8	0.08	N
PF00018	SH3_1	PF00071	Ras	PF00071	Ras	0.01	2	0	Y
PF00125	Histone	PF00125	Histone	PF03810	IBN_N	0.01	2	0.21	N
PF07690	MFS_1	PF00324	AA_permease	PF00674	PF00674	0.01	2	0.06	N
