# Supplementary material for: Organization of Physical Interactomes as Uncovered by Network Schemas
Source: PLoS Comput Biol. 2008 Oct 24;4(10):e1000203. doi: 10.1371/journal.pcbi.1000203 (PMC2561054; doi:10.1371/journal.pcbi.1000203)
Supplement: Table S5 — Emergent S. cerevisiae Pfam Y-star schemas (0.02 MB PDF) [file pcbi.1000203.s009.pdf]

Center ID	Center Name	ID_2	Name_2	ID_3	Name_3	ID_4	Name_4	FDR	Count in yeast	Avg count in random	Instance in human?
PF00069	Pkinase	PF00069	Pkinase	PF00096	zf-C2H2	PF00096	zf-C2H2	0	46	8.78	Y
PF00069	Pkinase	PF00069	Pkinase	PF00096	zf-C2H2	PF00172	PF00172	0	40	7.73	N
PF00069	Pkinase	PF00069	Pkinase	PF00134	Cyclin_N	PF00096	zf-C2H2	0	62	22.42	Y
PF00069	Pkinase	PF00069	Pkinase	PF00134	Cyclin_N	PF00621	RhoGEF	0	44	10.79	N
PF00069	Pkinase	PF00069	Pkinase	PF00134	Cyclin_N	PF00735	GTP_CDC	0.01	44	14.18	N
PF00069	Pkinase	PF00069	Pkinase	PF00096	zf-C2H2	PF00320	GATA	0.01	24	4.06	N
PF00069	Pkinase	PF00069	Pkinase	PF00096	zf-C2H2	PF00439	Bromodomain	0.01	20	3.27	Y
PF00018	SH3_1	PF00018	SH3_1	PF00069	Pkinase	PF02205	WH2	0.02	108	86.33	Y
PF00018	SH3_1	PF06371	Drf_GBD	PF00018	SH3_1	PF02205	WH2	0.02	60	40.52	N
PF00069	Pkinase	PF00018	SH3_1	PF00069	Pkinase	PF00564	PB1	0.02	27	8.9	Y
PF00069	Pkinase	PF00069	Pkinase	PF00621	RhoGEF	PF00735	GTP_CDC	0.02	14	2.07	N
PF00069	Pkinase	PF00069	Pkinase	PF00735	GTP_CDC	PF00735	GTP_CDC	0.02	16	2.91	N
PF00071	Ras	PF00069	Pkinase	PF00620	RhoGAP	PF00566	TBC	0.02	40	21.08	N
PF00018	SH3_1	PF06371	Drf_GBD	PF00018	SH3_1	PF00443	UCH	0.03	30	14.06	N
PF00069	Pkinase	PF00018	SH3_1	PF00069	Pkinase	PF00621	RhoGEF	0.03	25	9.67	Y
PF00069	Pkinase	PF00018	SH3_1	PF00069	Pkinase	PF00786	PBD	0.03	23	7.25	N
PF00069	Pkinase	PF00018	SH3_1	PF00071	Ras	PF00069	Pkinase	0.03	25	10.18	Y
PF00069	Pkinase	PF07647	SAM_2	PF00018	SH3_1	PF00564	PB1	0.03	26	10.94	Y
PF00071	Ras	PF00018	SH3_1	PF00620	RhoGAP	PF00620	RhoGAP	0.03	46	28.22	Y
PF00018	SH3_1	PF00022	Actin	PF00018	SH3_1	PF02205	WH2	0.04	44	30.05	N
PF00018	SH3_1	PF06371	Drf_GBD	PF00022	Actin	PF00018	SH3_1	0.04	34	19.12	N
PF00069	Pkinase	PF00018	SH3_1	PF00018	SH3_1	PF00400	WD40	0.04	23	10.47	Y
PF00069	Pkinase	PF00018	SH3_1	PF00069	Pkinase	PF00134	Cyclin_N	0.04	52	36.14	Y
PF00069	Pkinase	PF00018	SH3_1	PF00069	Pkinase	PF00169	PH	0.04	31	17.79	Y
PF00069	Pkinase	PF00018	SH3_1	PF00621	RhoGEF	PF00735	GTP_CDC	0.04	13	3.1	N
PF00069	Pkinase	PF00069	Pkinase	PF00069	Pkinase	PF00134	Cyclin_N	0.04	113	96.13	Y
PF00071	Ras	PF00069	Pkinase	PF00412	LIM	PF00566	TBC	0.04	21	8.84	N
PF00071	Ras	PF00069	Pkinase	PF00620	RhoGAP	PF00620	RhoGAP	0.04	51	35.57	Y
PF00071	Ras	PF00620	RhoGAP	PF00620	RhoGAP	PF00566	TBC	0.04	36	21.24	N
PF00071	Ras	PF00620	RhoGAP	PF00621	RhoGEF	PF00566	TBC	0.04	16	4.95	N
PF00069	Pkinase	PF00018	SH3_1	PF00069	Pkinase	PF00787	PX	0.05	14	4.22	N
PF01105	EMP24_GP25L	PF07690	MFS_1	PF00137	ATP-synt_C	PF00324	AA_permease	0.05	52	37.9	N
