# Supplementary material for: Organization of Physical Interactomes as Uncovered by Network Schemas
Source: PLoS Comput Biol. 2008 Oct 24;4(10):e1000203. doi: 10.1371/journal.pcbi.1000203 (PMC2561054; doi:10.1371/journal.pcbi.1000203)
Supplement: Table S6 — Emergent S. cerevisiae GO molecular function pair schemas (0.02 MB PDF) [file pcbi.1000203.s010.pdf]

ID_1	Name_1	ID_2	Name_2	FDR	Count in yeast	Avg count in random
GO:0003678	DNA helicase activity	GO:0003678	DNA helicase activity	0	9	0.61
GO:0003682	chromatin binding	GO:0003682	chromatin binding	0	11	0.18
GO:0003689	DNA clamp loader activity	GO:0003689	DNA clamp loader activity	0	8	0.06
GO:0003700	transcription factor activity	GO:0003702	RNA polymerase II transcription factor activity	0	20	7.95
GO:0003701	RNA polymerase I transcription factor activity	GO:0003701	RNA polymerase I transcription factor activity	0	8	0.12
GO:0003701	RNA polymerase I transcription factor activity	GO:0003702	RNA polymerase II transcription factor activity	0	16	3.82
GO:0003702	RNA polymerase II transcription factor activity	GO:0003702	RNA polymerase II transcription factor activity	0	105	14.76
GO:0003709	RNA polymerase III transcription factor activity	GO:0003709	RNA polymerase III transcription factor activity	0	8	0.11
GO:0003711	transcriptional elongation regulator activity	GO:0003711	transcriptional elongation regulator activity	0	8	0.17
GO:0003729	mRNA binding	GO:0003729	mRNA binding	0	10	1.21
GO:0003743	translation initiation factor activity	GO:0003743	translation initiation factor activity	0	42	0.8
GO:0003777	microtubule motor activity	GO:0005200	structural constituent of cytoskeleton	0	9	0.91
GO:0003779	actin binding	GO:0005200	structural constituent of cytoskeleton	0	12	1.92
GO:0003887	DNA-directed DNA polymerase activity	GO:0003887	DNA-directed DNA polymerase activity	0	24	0.48
GO:0003887	DNA-directed DNA polymerase activity	GO:0004536	deoxyribonuclease activity	0	8	0.92
GO:0003899	DNA-directed RNA polymerase activity	GO:0003899	DNA-directed RNA polymerase activity	0	41	1.2
GO:0003924	GTPase activity	GO:0005085	guanyl-nucleotide exchange factor activity	0	21	2.51
GO:0003924	GTPase activity	GO:0005092	GDP-dissociation inhibitor activity	0	14	0.59
GO:0003924	GTPase activity	GO:0005096	GTPase activator activity	0	44	3.71
GO:0003924	GTPase activity	GO:0051020	GTPase binding	0	15	1.24
GO:0004519	endonuclease activity	GO:0004519	endonuclease activity	0	28	1
GO:0004527	exonuclease activity	GO:0004527	exonuclease activity	0	17	0.57
GO:0004536	deoxyribonuclease activity	GO:0003684	damaged DNA binding	0	7	0.38
GO:0004540	ribonuclease activity	GO:0004540	ribonuclease activity	0	45	1.32
GO:0004672	protein kinase activity	GO:0003700	transcription factor activity	0	25	9.95
GO:0004672	protein kinase activity	GO:0004672	protein kinase activity	0	59	19.35
GO:0004672	protein kinase activity	GO:0005519	cytoskeletal regulatory protein binding	0	12	3.42
GO:0004672	protein kinase activity	GO:0019207	kinase regulator activity	0	66	15.44
GO:0004721	phosphoprotein phosphatase activity	GO:0004672	protein kinase activity	0	21	8.73
GO:0004721	phosphoprotein phosphatase activity	GO:0019208	phosphatase regulator activity	0	19	0.67
GO:0005057	receptor signaling protein activity	GO:0004672	protein kinase activity	0	25	7.8
GO:0005096	GTPase activator activity	GO:0005519	cytoskeletal regulatory protein binding	0	8	0.66
GO:0005200	structural constituent of cytoskeleton	GO:0005200	structural constituent of cytoskeleton	0	75	6.75
GO:0005275	amine transporter activity	GO:0008324	cation transporter activity	0	15	5.11
GO:0005275	amine transporter activity	GO:0008509	anion transporter activity	0	8	0.73
GO:0005275	amine transporter activity	GO:0016746	transferase activity transferring acyl groups	0	27	5.93
GO:0005275	amine transporter activity	GO:0016757	transferase activity transferring glycosyl groups	0	18	5.99
GO:0005275	amine transporter activity	GO:0042124	1 3-beta-glucanosyltransferase activity	0	9	1.29
GO:0005342	organic acid transporter activity	GO:0016746	transferase activity transferring acyl groups	0	24	5.75
GO:0005342	organic acid transporter activity	GO:0016757	transferase activity transferring glycosyl groups	0	16	5.7
GO:0005485	v-SNARE activity	GO:0000149	SNARE binding	0	10	0.36
GO:0005485	v-SNARE activity	GO:0005485	v-SNARE activity	0	29	1.03
GO:0005485	v-SNARE activity	GO:0005486	t-SNARE activity	0	34	1.47
GO:0008134	transcription factor binding	GO:0003702	RNA polymerase II transcription factor activity	0	40	10.73
GO:0008134	transcription factor binding	GO:0008134	transcription factor binding	0	25	2.04
GO:0008168	methyltransferase activity	GO:0008168	methyltransferase activity	0	10	0.42
GO:0008233	peptidase activity	GO:0003735	structural constituent of ribosome	0	10	2.01
GO:0008233	peptidase activity	GO:0008233	peptidase activity	0	35	2.27
GO:0008324	cation transporter activity	GO:0008324	cation transporter activity	0	31	3.23
GO:0008565	protein transporter activity	GO:0003700	transcription factor activity	0	13	4.25
GO:0008565	protein transporter activity	GO:0008565	protein transporter activity	0	33	3.84
GO:0008639	small protein conjugating enzyme activity	GO:0019787	ubiquitin-like-protein ligase activity	0	12	0.92
GO:0015631	tubulin binding	GO:0005200	structural constituent of cytoskeleton	0	22	2.28
GO:0016564	transcriptional repressor activity	GO:0003702	RNA polymerase II transcription factor activity	0	17	6.83
GO:0016564	transcriptional repressor activity	GO:0008134	transcription factor binding	0	17	2.27
GO:0016564	transcriptional repressor activity	GO:0016564	transcriptional repressor activity	0	14	0.67
GO:0016564	transcriptional repressor activity	GO:0019213	deacetylase activity	0	11	0.88
GO:0016746	transferase activity transferring acyl groups	GO:0015144	carbohydrate transporter activity	0	12	1.81
GO:0016746	transferase activity transferring acyl groups	GO:0016746	transferase activity transferring acyl groups	0	19	4.03
GO:0016746	transferase activity transferring acyl groups	GO:0016757	transferase activity transferring glycosyl groups	0	21	8.96
GO:0016757	transferase activity transferring glycosyl groups	GO:0008565	protein transporter activity	0	34	8.54
GO:0016757	transferase activity transferring glycosyl groups	GO:0016757	transferase activity transferring glycosyl groups	0	39	4.51
GO:0017022	myosin binding	GO:0000146	microfilament motor activity	0	9	0.15
GO:0019207	kinase regulator activity	GO:0019207	kinase regulator activity	0	17	2.45
GO:0019213	deacetylase activity	GO:0019213	deacetylase activity	0	11	0.11
GO:0019787	ubiquitin-like-protein ligase activity	GO:0019787	ubiquitin-like-protein ligase activity	0	18	0.92
GO:0030276	clathrin binding	GO:0030276	clathrin binding	0	8	0.07
GO:0031202	RNA splicing factor activity, transesterification mechanism	GO:0003724	RNA helicase activity	0	13	2.02
GO:0031202	RNA splicing factor activity, transesterification mechanism	GO:0003729	mRNA binding	0	19	5.66
GO:0031202	RNA splicing factor activity, transesterification mechanism	GO:0031202	RNA splicing factor activity, transesterification mechanism	0	60	5.01
GO:0031386	protein tag	GO:0008233	peptidase activity	0	12	1.66
GO:0042626	ATPase activity coupled to transmembrane movement of substances	GO:0008324	cation transporter activity	0	20	3
GO:0042626	ATPase activity coupled to transmembrane movement of substances	GO:0042626	ATPase activity coupled to transmembrane movement of substances	0	14	0.87
GO:0043565	sequence-specific DNA binding	GO:0008301	DNA bending activity	0	11	1.55
GO:0043565	sequence-specific DNA binding	GO:0019787	ubiquitin-like-protein ligase activity	0	18	2.84
GO:0043565	sequence-specific DNA binding	GO:0043565	sequence-specific DNA binding	0	25	1.56
GO:0043566	structure-specific DNA binding	GO:0043566	structure-specific DNA binding	0	7	0.44
GO:0051082	unfolded protein binding	GO:0008565	protein transporter activity	0	14	4.91
GO:0051082	unfolded protein binding	GO:0051082	unfolded protein binding	0	17	1.56
GO:0051082	unfolded protein binding	GO:0051087	chaperone binding	0	8	0.4
GO:0003689	DNA clamp loader activity	GO:0003887	DNA-directed DNA polymerase activity	0.01	6	0.36
GO:0003887	DNA-directed DNA polymerase activity	GO:0004527	exonuclease activity	0.01	8	1.23
GO:0003923	GPI-anchor transamidase activity	GO:0016746	transferase activity transferring acyl groups	0.01	8	1.27
GO:0004536	deoxyribonuclease activity	GO:0004536	deoxyribonuclease activity	0.01	6	0.29

GO:0004536	deoxyribonuclease activity	GO:0005524	ATP binding	0.01	6	0.48
GO:0004721	phosphoprotein phosphatase activity	GO:0004721	phosphoprotein phosphatase activity	0.01	8	1.08
GO:0005275	amine transporter activity	GO:0005342	organic acid transporter activity	0.01	9	2.45
GO:0005275	amine transporter activity	GO:0016722	oxidoreductase activity oxidizing metal ions	0.01	7	0.71
GO:0005342	organic acid transporter activity	GO:0008324	cation transporter activity	0.01	12	4.73
GO:0005342	organic acid transporter activity	GO:0008509	anion transporter activity	0.01	7	0.71
GO:0005486	t-SNARE activity	GO:0000149	SNARE binding	0.01	6	0.21
GO:0005519	cytoskeletal regulatory protein binding	GO:0005519	cytoskeletal regulatory protein binding	0.01	6	0.07
GO:0005524	ATP binding	GO:0005524	ATP binding	0.01	6	0.1
GO:0016757	transferase activity transferring glycosyl groups	GO:0008324	cation transporter activity	0.01	15	7.16
GO:0016757	transferase activity transferring glycosyl groups	GO:0042124	1 3-beta-glucanosyltransferase activity	0.01	8	1.52
GO:0016765	transferase activity transferring alkyl or aryl (other than methyl)	GO:0016765	transferase activity transferring alkyl or aryl (other than methyl) groups	0.01	5	0.06
GO:0030674	protein binding bridging	GO:0030674	protein binding bridging	0.01	7	0.51
GO:0031202	RNA splicing factor activity, transesterification mechanism	GO:0003735	structural constituent of ribosome	0.01	10	2.92
GO:0031386	protein tag	GO:0019787	ubiquitin-like-protein ligase activity	0.01	7	1.14
GO:0035091	phosphoinositide binding	GO:0035091	phosphoinositide binding	0.01	6	0.21
GO:0042626	ATPase activity coupled to transmembrane movement of substances	GO:0005275	amine transporter activity	0.01	10	3.03
GO:0042626	ATPase activity coupled to transmembrane movement of substances	GO:0005342	organic acid transporter activity	0.01	10	2.79
GO:0051082	unfolded protein binding	GO:0030188	chaperone regulator activity	0.01	6	0.55
GO:0003678	DNA helicase activity	GO:0003684	damaged DNA binding	0.02	6	0.75
GO:0003684	damaged DNA binding	GO:0003684	damaged DNA binding	0.02	5	0.1
GO:0003702	RNA polymerase II transcription factor activity	GO:0003678	DNA helicase activity	0.02	14	6.78
GO:0003724	RNA helicase activity	GO:0003724	RNA helicase activity	0.02	5	0.18
GO:0003735	structural constituent of ribosome	GO:0003735	structural constituent of ribosome	0.02	5	0.33
GO:0003779	actin binding	GO:0003779	actin binding	0.02	5	0.12
GO:0003899	DNA-directed RNA polymerase activity	GO:0003702	RNA polymerase II transcription factor activity	0.02	16	8.94
GO:0004497	monooxygenase activity	GO:0005275	amine transporter activity	0.02	6	0.9
GO:0004497	monooxygenase activity	GO:0005342	organic acid transporter activity	0.02	6	0.91
GO:0004497	monooxygenase activity	GO:0016746	transferase activity transferring acyl groups	0.02	7	1.43
GO:0004672	protein kinase activity	GO:0008134	transcription factor binding	0.02	20	11.94
GO:0004672	protein kinase activity	GO:0035091	phosphoinositide binding	0.02	11	4.4
GO:0005486	t-SNARE activity	GO:0005486	t-SNARE activity	0.02	5	0.27
GO:0008168	methyltransferase activity	GO:0030515	snoRNA binding	0.02	5	0.17
GO:0008301	DNA bending activity	GO:0003702	RNA polymerase II transcription factor activity	0.02	11	4.63
GO:0016722	oxidoreductase activity oxidizing metal ions	GO:0008324	cation transporter activity	0.02	6	0.9
GO:0003700	transcription factor activity	GO:0003700	transcription factor activity	0.03	6	1.09
GO:0003724	RNA helicase activity	GO:0003729	mRNA binding	0.03	6	1.13
GO:0004527	exonuclease activity	GO:0003702	RNA polymerase II transcription factor activity	0.03	12	5.82
GO:0005200	structural constituent of cytoskeleton	GO:0000146	microfilament motor activity	0.03	8	2.38
GO:0016746	transferase activity transferring acyl groups	GO:0042124	1 3-beta-glucanosyltransferase activity	0.03	7	1.78
GO:0031386	protein tag	GO:0008639	small protein conjugating enzyme activity	0.03	5	0.46
GO:0003746	translation elongation factor activity	GO:0003746	translation elongation factor activity	0.04	4	0.02
GO:0003899	DNA-directed RNA polymerase activity	GO:0003709	RNA polymerase III transcription factor activity	0.04	6	1.16
GO:0004749	ribose phosphate diphosphokinase activity	GO:0004749	ribose phosphate diphosphokinase activity	0.04	4	0.01
GO:0005200	structural constituent of cytoskeleton	GO:0043565	sequence-specific DNA binding	0.04	13	6.76
GO:0008324	cation transporter activity	GO:0042124	1 3-beta-glucanosyltransferase activity	0.04	6	1.28
GO:0016667	oxidoreductase activity acting on sulfur group of donors	GO:0015036	disulfide oxidoreductase activity	0.04	4	0.04
GO:0016746	transferase activity transferring acyl groups	GO:0008134	transcription factor binding	0.04	12	5.92
GO:0016746	transferase activity transferring acyl groups	GO:0051184	cofactor transporter activity	0.04	6	1.31
GO:0019207	kinase regulator activity	GO:0003700	transcription factor activity	0.04	9	3.56
GO:0043566	structure-specific DNA binding	GO:0008639	small protein conjugating enzyme activity	0.04	5	0.61
GO:0008134	transcription factor binding	GO:0003700	transcription factor activity	0.05	8	2.81
GO:0031386	protein tag	GO:0030674	protein binding bridging	0.05	5	0.67
GO:0031386	protein tag	GO:0043130	ubiquitin binding	0.05	4	0.09
