# Supplementary material for: Organization of Physical Interactomes as Uncovered by Network Schemas
Source: PLoS Comput Biol. 2008 Oct 24;4(10):e1000203. doi: 10.1371/journal.pcbi.1000203 (PMC2561054; doi:10.1371/journal.pcbi.1000203)
Supplement: Table S7 — Emergent H. sapiens Pfam pair schemas (0.05 MB PDF) [file pcbi.1000203.s011.pdf]

ID_1	Name_1	ID_2	Name_2	FDR	Count in human	Average count in random
PF00001	7tm_1	PF00001	7tm_1	0	14	2.21
PF00001	7tm_1	PF00048	IL8	0	37	1.25
PF00001	7tm_1	PF00339	Arrestin_N	0	9	0.61
PF00001	7tm_1	PF00503	G-alpha	0	63	2.36
PF00001	7tm_1	PF00615	RGS	0	10	1.55
PF00001	7tm_1	PF03002	Somatostatin	0	12	0.18
PF00008	EGF	PF00008	EGF	0	22	3.65
PF00008	EGF	PF00040	fn2	0	9	1.36
PF00008	EGF	PF00041	fn3	0	20	7.06
PF00008	EGF	PF00047	ig	0	16	5.79
PF00008	EGF	PF00053	Laminin_EGF	0	8	0.84
PF00008	EGF	PF00079	Serpin	0	9	1.92
PF00008	EGF	PF00089	Trypsin	0	14	3.32
PF00008	EGF	PF00147	Fibrinogen_C	0	8	0.69
PF00008	EGF	PF00413	Peptidase_M10	0	18	1.08
PF00008	EGF	PF00640	PID	0	16	2.55
PF00008	EGF	PF01391	Collagen	0	19	2.85
PF00008	EGF	PF01821	ANATO	0	9	0.39
PF00008	EGF	PF02210	Laminin_G_2	0	10	1.25
PF00008	EGF	PF07645	EGF_CA	0	24	3.61
PF00008	EGF	PF07679	I-set	0	12	3.33
PF00008	EGF	PF07974	EGF_2	0	14	2.32
PF00010	HLH	PF00010	HLH	0	34	1.09
PF00010	HLH	PF00104	Hormone_recep	0	25	3.58
PF00010	HLH	PF00439	Bromodomain	0	9	1.75
PF00012	HSP70	PF00515	TPR_1	0	7	0.34
PF00013	KH_1	PF00013	KH_1	0	6	0.1
PF00013	KH_1	PF00076	RRM_1	0	13	1.14
PF00017	SH2	PB001482	Pfam-B_1482	0	8	0.43
PF00017	SH2	PB023385	Pfam-B_23385	0	6	0.28
PF00017	SH2	PF00017	SH2	0	79	17.08
PF00017	SH2	PF00018	SH3_1	0	90	29.23
PF00017	SH2	PF00041	fn3	0	73	15.78
PF00017	SH2	PF00047	ig	0	62	12.8
PF00017	SH2	PF00097	zf-C3HC4	0	25	13.47
PF00017	SH2	PF00102	Y_phosphatase	0	28	6.08
PF00017	SH2	PF00130	C1_1	0	20	9.26
PF00017	SH2	PF00169	PH	0	49	14.92
PF00017	SH2	PF00620	RhoGAP	0	16	4.36
PF00017	SH2	PF00621	RhoGEF	0	17	5.59
PF00017	SH2	PF00627	UBA	0	18	3.76
PF00017	SH2	PF02174	IRS	0	13	1.82
PF00017	SH2	PF02189	ITAM	0	9	1.28
PF00017	SH2	PF07686	V-set	0	28	7.38
PF00017	SH2	PF07714	Pkinase_Tyr	0	168	28.13
PF00018	SH3_1	PF00018	SH3_1	0	47	16.75
PF00018	SH3_1	PF00041	fn3	0	40	16.02
PF00018	SH3_1	PF00047	ig	0	40	12.4
PF00018	SH3_1	PF00097	zf-C3HC4	0	24	13.38
PF00018	SH3_1	PF00102	Y_phosphatase	0	18	6.14
PF00018	SH3_1	PF00169	PH	0	42	14.85

PF00018	SH3_1	PF00350	Dynamin_N	0	8	0.61
PF00018	SH3_1	PF00412	LIM	0	16	7.34
PF00018	SH3_1	PF00568	WH1	0	15	1.85
PF00018	SH3_1	PF00617	RasGEF	0	12	1.54
PF00018	SH3_1	PF00620	RhoGAP	0	12	4.27
PF00018	SH3_1	PF00621	RhoGEF	0	14	5.53
PF00018	SH3_1	PF00627	UBA	0	21	3.79
PF00018	SH3_1	PF00786	PBD	0	14	1.69
PF00018	SH3_1	PF02189	ITAM	0	8	1.32
PF00018	SH3_1	PF02205	WH2	0	16	1.15
PF00018	SH3_1	PF07686	V-set	0	16	7.35
PF00018	SH3_1	PF07714	Pkinase_Tyr	0	113	29.19
PF00019	TGF_beta	PF00019	TGF_beta	0	7	0.21
PF00019	TGF_beta	PF00069	Pkinase	0	42	5.31
PF00019	TGF_beta	PF00100	Zona_pellucida	0	7	0.14
PF00020	TNFR_c6	PF00097	zf-C3HC4	0	13	1.32
PF00020	TNFR_c6	PF00229	TNF	0	17	0.22
PF00020	TNFR_c6	PF00917	MATH	0	14	0.3
PF00022	Actin	PF00241	Cofilin_ADF	0	9	0.12
PF00022	Actin	PF00435	Spectrin	0	7	0.51
PF00023	Ank	PF00023	Ank	0	9	1.03
PF00023	Ank	PF00069	Pkinase	0	22	10.88
PF00023	Ank	PF00169	PH	0	12	4.25
PF00023	Ank	PF01833	TIG	0	12	1.1
PF00023	Ank	PF07679	I-set	0	10	1.92
PF00025	Arf	PF06456	Arfaptin	0	6	0.05
PF00028	Cadherin	PF00514	Arm	0	15	0.7
PF00031	Cystatin	PF00112	Peptidase_C1	0	7	0.02
PF00036	efhand	PF00036	efhand	0	20	1.82
PF00036	efhand	PF00520	Ion_trans	0	12	1.78
PF00036	efhand	PF00622	SPRY	0	7	0.77
PF00036	efhand	PF01023	S_100	0	8	0.54
PF00038	Filament	PF00038	Filament	0	16	0.56
PF00038	Filament	PF00244	14-3-3	0	7	0.82
PF00038	Filament	PF00681	Plectin	0	7	0.25
PF00040	fn2	PF01391	Collagen	0	9	0.48
PF00041	fn3	PF00041	fn3	0	20	3.6
PF00041	fn3	PF00047	ig	0	21	6.05
PF00041	fn3	PF00102	Y_phosphatase	0	19	2.62
PF00041	fn3	PF00169	PH	0	23	7.59
PF00041	fn3	PF00812	Ephrin	0	9	0.37
PF00041	fn3	PF02174	IRS	0	8	0.88
PF00041	fn3	PF07679	I-set	0	15	3.3
PF00041	fn3	PF07686	V-set	0	11	3.28
PF00041	fn3	PF07714	Pkinase_Tyr	0	33	13.98
PF00045	Hemopexin	PF00965	TIMP	0	8	0.06
PF00045	Hemopexin	PF07686	V-set	0	7	0.6
PF00046	Homeobox	PF00046	Homeobox	0	28	1.23
PF00046	Homeobox	PF00104	Hormone_recep	0	15	3.31
PF00046	Homeobox	PF00292	PAX	0	6	0.23
PF00047	ig	PF00047	ig	0	15	2.5
PF00047	ig	PF00102	Y_phosphatase	0	12	2.29
PF00047	ig	PF00167	FGF	0	9	0.35

PF00047	ig	PF00640	PID	0	9	1.92
PF00047	ig	PF07645	EGF_CA	0	11	3.18
PF00047	ig	PF07679	I-set	0	13	2.55
PF00047	ig	PF07686	V-set	0	15	2.83
PF00047	ig	PF07714	Pkinase_Tyr	0	30	11.29
PF00048	IL8	PF07686	V-set	0	7	0.71
PF00059	Lectin_C	PF00059	Lectin_C	0	8	0.22
PF00059	Lectin_C	PF00084	Sushi	0	7	0.54
PF00060	Lig_chan	PF00595	PDZ	0	21	1.28
PF00069	Pkinase	PF00069	Pkinase	0	98	24.58
PF00069	Pkinase	PF00130	C1_1	0	34	10.61
PF00069	Pkinase	PF00134	Cyclin_N	0	17	4.74
PF00069	Pkinase	PF00169	PH	0	31	19.32
PF00069	Pkinase	PF00244	14-3-3	0	20	5.85
PF00069	Pkinase	PF00531	Death	0	20	7.78
PF00069	Pkinase	PF00564	PB1	0	16	3.28
PF00069	Pkinase	PF00581	Rhodanese	0	15	1.93
PF00069	Pkinase	PF00917	MATH	0	13	4.3
PF00069	Pkinase	PF01412	ArfGap	0	10	1.83
PF00071	Ras	PB000002	Pfam-B_2	0	21	3.72
PF00071	Ras	PF00130	C1_1	0	13	3.46
PF00071	Ras	PF00168	C2	0	16	4.17
PF00071	Ras	PF00169	PH	0	25	6.4
PF00071	Ras	PF00595	PDZ	0	19	8.08
PF00071	Ras	PF00617	RasGEF	0	12	0.58
PF00071	Ras	PF00620	RhoGAP	0	11	1.74
PF00071	Ras	PF00621	RhoGEF	0	14	1.97
PF00071	Ras	PF00786	PBD	0	12	0.72
PF00071	Ras	PF00788	RA	0	26	1.2
PF00071	Ras	PF01843	DIL	0	12	0.59
PF00071	Ras	PF02115	Rho_GDI	0	8	0.17
PF00071	Ras	PF02204	VPS9	0	7	0.13
PF00071	Ras	PF06920	Ded_cyto	0	6	0.2
PF00076	RRM_1	PF00076	RRM_1	0	40	1.77
PF00076	RRM_1	PF00397	WW	0	9	1.64
PF00079	Serpin	PF00089	Trypsin	0	47	0.79
PF00084	Sushi	PF00084	Sushi	0	6	0.27
PF00084	Sushi	PF00147	Fibrinogen_C	0	6	0.25
PF00084	Sushi	PF01821	ANATO	0	9	0.18
PF00089	Trypsin	PF00089	Trypsin	0	7	0.7
PF00089	Trypsin	PF01391	Collagen	0	10	1.5
PF00092	VWA	PF01391	Collagen	0	9	0.51
PF00096	zf-C2H2	PF00096	zf-C2H2	0	28	2.1
PF00096	zf-C2H2	PF00097	zf-C3HC4	0	14	4.35
PF00096	zf-C2H2	PF00643	zf-B_box	0	11	1.44
PF00096	zf-C2H2	PF00651	BTB	0	11	1.03
PF00096	zf-C2H2	PF02023	SCAN	0	20	0.78
PF00096	zf-C2H2	PF03165	MH1	0	10	1.14
PF00097	zf-C3HC4	PF00179	UQ_con	0	18	1.08
PF00097	zf-C3HC4	PF00888	Cullin	0	6	0.28
PF00097	zf-C3HC4	PF01454	MAGE	0	8	0.92
PF00102	Y_phosphatase	PF00169	PH	0	12	2.61
PF00102	Y_phosphatase	PF07714	Pkinase_Tyr	0	28	5.25

PF00104	Hormone_recep	PF00104	Hormone_recep	0	31	2.19
PF00104	Hormone_recep	PF00249	Myb_DNA-binding	0	9	1.42
PF00104	Hormone_recep	PF00439	Bromodomain	0	17	1.97
PF00104	Hormone_recep	PF00628	PHD	0	9	0.96
PF00104	Hormone_recep	PF00989	PAS	0	32	1.92
PF00110	wnt	PF01392	Fz	0	7	0
PF00128	Alpha-amylase	PF00324	AA_permease	0	6	0
PF00130	C1_1	PF00169	PH	0	19	4.24
PF00130	C1_1	PF00244	14-3-3	0	11	1.22
PF00130	C1_1	PF07714	Pkinase_Tyr	0	18	7.95
PF00168	C2	PF00169	PH	0	15	4.71
PF00168	C2	PF07714	Pkinase_Tyr	0	23	9.8
PF00169	PH	PF00169	PH	0	12	3.5
PF00169	PH	PF01412	ArfGap	0	7	0.63
PF00169	PH	PF07714	Pkinase_Tyr	0	60	13.49
PF00170	bZIP_1	PB001741	Pfam-B_1741	0	8	0.16
PF00170	bZIP_1	PF00170	bZIP_1	0	8	0.21
PF00170	bZIP_1	PF07716	bZIP_2	0	16	0.36
PF00179	UQ_con	PF00632	HECT	0	8	0.19
PF00179	UQ_con	PF01485	IBR	0	8	0.07
PF00193	Xlink	PF00413	Peptidase_M10	0	8	0.16
PF00307	CH	PF00412	LIM	0	12	1.67
PF00341	PDGF	PF07679	I-set	0	7	0.19
PF00373	Band_41	PB000002	Pfam-B_2	0	8	1.14
PF00373	Band_41	PF00595	PDZ	0	10	2.14
PF00400	WD40	PF00631	G-gamma	0	15	0.24
PF00452	Bcl-2	PF00452	Bcl-2	0	13	0.16
PF00503	G-alpha	PF00615	RGS	0	24	0.8
PF00503	G-alpha	PF02188	GoLoco	0	8	0.21
PF00520	Ion_trans	PF00520	Ion_trans	0	30	0.46
PF00531	Death	PF00531	Death	0	12	0.38
PF00531	Death	PF00656	Peptidase_C14	0	8	0.49
PF00531	Death	PF00917	MATH	0	8	0.63
PF00531	Death	PF01335	DED	0	13	0.31
PF00531	Death	PF01582	TIR	0	7	0.22
PF00560	LRR_1	PF00560	LRR_1	0	7	0.77
PF00560	LRR_1	PF00619	CARD	0	7	0.8
PF00564	PB1	PF00564	PB1	0	7	0.05
PF00595	PDZ	PF00595	PDZ	0	16	5.38
PF00619	CARD	PF00619	CARD	0	14	0.18
PF00619	CARD	PF00656	Peptidase_C14	0	17	0.48
PF00619	CARD	PF05729	NACHT	0	7	0.03
PF00620	RhoGAP	PF07714	Pkinase_Tyr	0	19	4.25
PF00621	RhoGEF	PF07714	Pkinase_Tyr	0	14	5.09
PF00627	UBA	PF07714	Pkinase_Tyr	0	13	3.18
PF00640	PID	PF02177	A4_EXTRA	0	9	0.2
PF00640	PID	PF07714	Pkinase_Tyr	0	15	4.66
PF00653	BIR	PF00656	Peptidase_C14	0	7	0.22
PF00656	Peptidase_C14	PF00656	Peptidase_C14	0	6	0.23
PF00656	Peptidase_C14	PF01335	DED	0	9	0.23
PF00735	GTP_CDC	PF00735	GTP_CDC	0	6	0.01
PF00754	F5_F8_type_C	PF01391	Collagen	0	6	0.2
PF00787	PX	PF07714	Pkinase_Tyr	0	10	2.1

PF00788	RA	PF07714	Pkinase_Tyr	0	15	2.69
PF00822	PMP22_Claudin	PF07653	SH3_2	0	6	0.12
PF00850	Hist_deacetyl	PF00850	Hist_deacetyl	0	7	0.19
PF00957	Synaptobrevin	PF05739	SNARE	0	12	0.15
PF00989	PAS	PB000064	Pfam-B_64	0	8	0.11
PF00989	PAS	PB003013	Pfam-B_3013	0	7	0.06
PF00989	PAS	PF00989	PAS	0	14	0.3
PF01007	IRK	PF00595	PDZ	0	11	0.4
PF01034	Syndecan	PF00595	PDZ	0	7	0.52
PF01094	ANF_receptor	PF00595	PDZ	0	20	1.62
PF01094	ANF_receptor	PF01094	ANF_receptor	0	6	0.16
PF01217	Clat_adaptor_s	PF01602	Adaptin_N	0	7	0.01
PF01335	DED	PF01335	DED	0	8	0.06
PF01391	Collagen	PF01391	Collagen	0	7	0.67
PF01391	Collagen	PF01462	LRRNT	0	11	0.54
PF01391	Collagen	PF07645	EGF_CA	0	13	1.56
PF01423	LSM	PF01423	LSM	0	20	0.19
PF01839	FG-GAP	PF03921	ICAM_N	0	7	0.14
PF01839	FG-GAP	PF07974	EGF_2	0	16	0.65
PF02071	NSF	PF05739	SNARE	0	8	0.09
PF02174	IRS	PF07714	Pkinase_Tyr	0	15	1.47
PF02197	RIIa	PF05716	AKAP_110	0	7	0.03
PF02210	Laminin_G_2	PF07714	Pkinase_Tyr	0	10	2.33
PF02319	E2F_TDP	PF02319	E2F_TDP	0	7	0.03
PF04857	CAF1	PF07742	BTG	0	6	0
PF05739	SNARE	PF05739	SNARE	0	12	0.17
PF07645	EGF_CA	PF07679	I-set	0	9	1.94
PF07654	C1-set	PF07654	C1-set	0	11	0.15
PF07679	I-set	PF07679	I-set	0	12	0.61
PF07686	V-set	PF07686	V-set	0	12	0.77
PF07714	Pkinase_Tyr	PF07714	Pkinase_Tyr	0	50	13.37
PF00002	7tm_2	PF04901	RAMP	0.01	5	0.03
PF00005	ABC_tran	PF00595	PDZ	0.01	7	1.26
PF00008	EGF	PF00048	IL8	0.01	7	1.34
PF00008	EGF	PF07714	Pkinase_Tyr	0.01	23	13.85
PF00008	EGF	PF07732	Cu-oxidase_3	0.01	5	0.26
PF00017	SH2	PF00028	Cadherin	0.01	10	3.57
PF00017	SH2	PF01146	Caveolin	0.01	7	1.49
PF00018	SH3_1	PF00536	SAM_1	0.01	9	2.77
PF00022	Actin	PF00307	CH	0.01	7	0.99
PF00022	Actin	PF00626	Gelsolin	0.01	5	0.11
PF00023	Ank	PF00041	fn3	0.01	10	3.7
PF00023	Ank	PF00615	RGS	0.01	6	0.93
PF00025	Arf	PF00169	PH	0.01	6	0.67
PF00036	efhand	PF01080	Presenilin	0.01	6	0.55
PF00041	fn3	PF00049	Insulin	0.01	5	0.29
PF00041	fn3	PF00059	Lectin_C	0.01	8	1.97
PF00041	fn3	PF00193	Xlink	0.01	7	0.88
PF00041	fn3	PF07645	EGF_CA	0.01	11	3.99
PF00047	ig	PF00791	ZU5	0.01	6	0.41
PF00047	ig	PF07654	C1-set	0.01	7	1.31
PF00047	ig	PF07974	EGF_2	0.01	8	2.18
PF00050	Kazal_1	PF00089	Trypsin	0.01	5	0.12

PF00057	Ldl_recept_a	PF01821	ANATO	0.01	5	0.08
PF00060	Lig_chan	PF00060	Lig_chan	0.01	5	0.07
PF00069	Pkinase	PF01248	Ribosomal_L7Ae	0.01	6	0.58
PF00069	Pkinase	PF01387	Synuclein	0.01	6	0.95
PF00071	Ras	PF00996	GDI	0.01	5	0.09
PF00071	Ras	PF02196	RBD	0.01	7	0.93
PF00076	RRM_1	PF00641	zf-RanBP	0.01	6	0.79
PF00084	Sushi	PF07974	EGF_2	0.01	6	0.78
PF00089	Trypsin	PF00594	Gla	0.01	5	0.34
PF00090	TSP_1	PF00413	Peptidase_M10	0.01	5	0.12
PF00096	zf-C2H2	PF00389	2-Hacid_dh	0.01	6	0.34
PF00096	zf-C2H2	PF00628	PHD	0.01	6	0.91
PF00097	zf-C3HC4	PF00097	zf-C3HC4	0.01	8	2.25
PF00097	zf-C3HC4	PF00385	Chromo	0.01	6	0.71
PF00102	Y_phosphatase	PF02174	IRS	0.01	6	0.41
PF00134	Cyclin_N	PF01857	RB_B	0.01	5	0.31
PF00179	UQ_con	PF00240	ubiquitin	0.01	5	0.32
PF00194	Carb_anhydrase	PF00955	HCO3_cotransp	0.01	5	0.02
PF00240	ubiquitin	PF02809	UIM	0.01	5	0.23
PF00170	bZIP_1	PF03131	bZIP_Maf	0.01	5	0.03
PF00249	Myb_DNA-binding	PF00850	Hist_deacetyl	0.01	6	0.49
PF00307	CH	PF05556	Calsarcin	0.01	5	0.1
PF00307	CH	PF07679	I-set	0.01	8	1.53
PF00335	Tetraspannin	PF00335	Tetraspannin	0.01	5	0.01
PF00340	IL1	PF01582	TIR	0.01	5	0.07
PF00412	LIM	PF00412	LIM	0.01	6	0.89
PF00431	CUB	PF01391	Collagen	0.01	5	0.16
PF00503	G-alpha	PF00621	RhoGEF	0.01	6	0.94
PF00505	HMG_box	PF00514	Arm	0.01	6	0.49
PF00514	Arm	PF00514	Arm	0.01	6	0.68
PF00515	TPR_1	PF00515	TPR_1	0.01	5	0.24
PF00520	Ion_trans	PF02060	ISK_Channel	0.01	5	0.03
PF00531	Death	PF00619	CARD	0.01	6	0.7
PF00560	LRR_1	PF01463	LRRCT	0.01	5	0.24
PF00560	LRR_1	PF01582	TIR	0.01	5	0.35
PF00594	Gla	PF07732	Cu-oxidase_3	0.01	5	0.03
PF00619	CARD	PF02758	PAAD_DAPIN	0.01	5	0.01
PF00621	RhoGEF	PF01403	Sema	0.01	5	0.12
PF00808	CBFD_NFYB_HMF	PF00808	CBFD_NFYB_HMF	0.01	5	0.01
PF00850	Hist_deacetyl	PF02671	PAH	0.01	5	0.13
PF00928	Adap_comp_sub	PF02883	Alpha_adaptinC2	0.01	5	0.07
PF01217	Clat_adaptor_s	PF02883	Alpha_adaptinC2	0.01	5	0.01
PF01391	Collagen	PF01839	FG-GAP	0.01	6	0.91
PF01412	ArfGap	PB000002	Pfam-B_2	0.01	5	0.33
PF01821	ANATO	PF07645	EGF_CA	0.01	5	0.2
PF02931	Neur_chan_LBD	PF02931	Neur_chan_LBD	0.01	5	0
PF03165	MH1	PF03165	MH1	0.01	5	0.12
PF05008	V-SNARE	PF05739	SNARE	0.01	5	0.06
PF05739	SNARE	PB000002	Pfam-B_2	0.01	7	0.96
PF07645	EGF_CA	PF07974	EGF_2	0.01	7	1.38
PF07647	SAM_2	PB000002	Pfam-B_2	0.01	7	0.99
PF07686	V-set	PF07974	EGF_2	0.01	7	1.21
PF07714	Pkinase_Tyr	PF07686	V-set	0.01	14	6.28

PF00005	ABC_tran	PF00005	ABC_tran	0.02	4	0.02
PF00008	EGF	PF00262	Calreticulin	0.02	5	0.59
PF00013	KH_1	PF00018	SH3_1	0.02	9	3.47
PF00017	SH2	PF00168	C2	0.02	18	10.92
PF00018	SH3_1	PF00611	FCH	0.02	7	1.94
PF00023	Ank	PF00412	LIM	0.02	7	1.88
PF00025	Arf	PF05351	GMP_PDE_delta	0.02	4	0.01
PF00038	Filament	PF00130	C1_1	0.02	7	1.69
PF00040	fn2	PF01839	FG-GAP	0.02	5	0.37
PF00047	ig	PF02189	ITAM	0.02	5	0.53
PF00053	Laminin_EGF	PF07645	EGF_CA	0.02	5	0.55
PF00071	Ras	PF00564	PB1	0.02	6	1.16
PF00071	Ras	PF02185	HR1	0.02	5	0.53
PF00076	RRM_1	PF00271	Helicase_C	0.02	6	1.18
PF00084	Sushi	PF07645	EGF_CA	0.02	6	1.19
PF00090	TSP_1	PF00089	Trypsin	0.02	5	0.53
PF00102	Y_phosphatase	PF07647	SAM_2	0.02	5	0.56
PF00130	C1_1	PF00130	C1_1	0.02	6	1.18
PF00244	14-3-3	PF00850	Hist_deacetyl	0.02	5	0.55
PF00249	Myb_DNA-binding	PF00439	Bromodomain	0.02	5	0.6
PF00307	CH	PF07974	EGF_2	0.02	6	1.14
PF00571	CBS	PF04739	AMPKBI	0.02	4	0.01
PF00626	Gelsolin	PF00626	Gelsolin	0.02	4	0.01
PF00635	Motile_Sperm	PF00957	Synaptobrevin	0.02	4	0
PF00640	PID	PF07679	I-set	0.02	6	1.15
PF01652	IF4E	PF05456	eIF_4EBP	0.02	4	0
PF02136	NTF2	PB006713	Pfam-B_6713	0.02	4	0
PF00004	AAA	PF00004	AAA	0.03	4	0.05
PF00008	EGF	PF00014	Kunitz_BPTI	0.03	5	0.83
PF00008	EGF	PF00093	VWC	0.03	5	0.7
PF00010	HLH	PF00412	LIM	0.03	7	2.15
PF00012	HSP70	PF00226	DnaJ	0.03	4	0.06
PF00012	HSP70	PF00240	ubiquitin	0.03	4	0.2
PF00012	HSP70	PF02179	BAG	0.03	4	0.1
PF00014	Kunitz_BPTI	PF00040	fn2	0.03	4	0.14
PF00017	SH2	PF07679	I-set	0.03	14	7.98
PF00019	TGF_beta	PF00093	VWC	0.03	4	0.13
PF00019	TGF_beta	PF07974	EGF_2	0.03	5	0.68
PF00025	Arf	PF02883	Alpha_adaptinC2	0.03	4	0.09
PF00038	Filament	PF01477	PLAT	0.03	4	0.12
PF00038	Filament	PF02185	HR1	0.03	4	0.2
PF00041	fn3	PF00084	Sushi	0.03	7	2.26
PF00041	fn3	PF00621	RhoGEF	0.03	8	2.83
PF00041	fn3	PF01291	LIF_OSM	0.03	4	0.1
PF00046	Homeobox	PF00505	HMG_box	0.03	5	0.69
PF00046	Homeobox	PF02037	SAP	0.03	5	0.68
PF00047	ig	PF00048	IL8	0.03	6	1.53
PF00053	Laminin_EGF	PF00053	Laminin_EGF	0.03	4	0.07
PF00057	Ldl_recept_a	PF00207	A2M	0.03	4	0.12
PF00058	Ldl_recept_b	PF00595	PDZ	0.03	7	2.21
PF00059	Lectin_C	PF00129	MHC_I	0.03	4	0.21
PF00059	Lectin_C	PF00431	CUB	0.03	4	0.09
PF00059	Lectin_C	PF07645	EGF_CA	0.03	6	1.28

PF00060	Lig_chan	PF00373	Band_41	0.03	4	0.23
PF00084	Sushi	PF01391	Collagen	0.03	5	0.82
PF00089	Trypsin	PF02210	Laminin_G_2	0.03	5	0.63
PF00089	Trypsin	PF07645	EGF_CA	0.03	7	2.04
PF00089	Trypsin	PF07732	Cu-oxidase_3	0.03	4	0.14
PF00096	zf-C2H2	PF00610	DEP	0.03	5	0.84
PF00102	Y_phosphatase	PF02985	HEAT	0.03	5	0.79
PF00104	Hormone_recep	PF00240	ubiquitin	0.03	6	1.42
PF00104	Hormone_recep	PF00643	zf-B_box	0.03	6	1.39
PF00147	Fibrinogen_C	PF01839	FG-GAP	0.03	4	0.2
PF00168	C2	PF05739	SNARE	0.03	6	1.32
PF00169	PH	PF00620	RhoGAP	0.03	7	2.2
PF00178	Ets	PF07716	bZIP_2	0.03	4	0.15
PF00219	IGFBP	PF07974	EGF_2	0.03	4	0.15
PF00243	NGF	PF07679	I-set	0.03	4	0.07
PF00320	GATA	PB003900	Pfam-B_3900	0.03	4	0.03
PF00335	Tetraspannin	PF07974	EGF_2	0.03	4	0.2
PF00439	Bromodomain	PF05030	SSXT	0.03	4	0.06
PF00452	Bcl-2	PF06553	BNIP3	0.03	4	0.03
PF00503	G-alpha	PF00595	PDZ	0.03	9	3.62
PF00520	Ion_trans	PF00595	PDZ	0.03	8	3.04
PF00531	Death	PF00560	LRR_1	0.03	6	1.26
PF00536	SAM_1	PF00536	SAM_1	0.03	4	0.03
PF00536	SAM_1	PF07647	SAM_2	0.03	4	0.15
PF00595	PDZ	PF03165	MH1	0.03	7	2.06
PF00595	PDZ	PF07653	SH3_2	0.03	8	2.83
PF00605	IRF	PF01833	TIG	0.03	4	0.07
PF00619	CARD	PF00653	BIR	0.03	4	0.16
PF00638	Ran_BP1	PF03810	IBN_N	0.03	4	0.09
PF00995	Sec1	PF05739	SNARE	0.03	4	0.05
PF01391	Collagen	PF07546	EMI	0.03	4	0.08
PF01582	TIR	PF01582	TIR	0.03	4	0.07
PF01602	Adaptin_N	PF01602	Adaptin_N	0.03	4	0.05
PF01833	TIG	PF01833	TIG	0.03	4	0.23
PF01857	RB_B	PB000002	Pfam-B_2	0.03	5	0.61
PF02319	E2F_TDP	PF01857	RB_B	0.03	4	0.09
PF02437	Ski_Sno	PF03165	MH1	0.03	4	0.1
PF02985	HEAT	PB000002	Pfam-B_2	0.03	6	1.35
PF03810	IBN_N	PB020723	Pfam-B_20723	0.03	4	0.05
PF03921	ICAM_N	PF07974	EGF_2	0.03	4	0.13
PF05507	MAGP	PF07645	EGF_CA	0.03	4	0.08
PF07716	bZIP_2	PF07716	bZIP_2	0.03	4	0.11
PF00010	HLH	PF00319	SRF-TF	0.04	4	0.32
PF00013	KH_1	PF00017	SH2	0.04	8	3.24
PF00017	SH2	PB001239	Pfam-B_1239	0.04	4	0.31
PF00018	SH3_1	PF07653	SH3_2	0.04	10	5.09
PF00019	TGF_beta	PF01462	LRRNT	0.04	4	0.35
PF00040	fn2	PF00048	IL8	0.04	4	0.3
PF00040	fn2	PF07645	EGF_CA	0.04	5	0.89
PF00046	Homeobox	PF00439	Bromodomain	0.04	6	1.7
PF00046	Homeobox	PF03920	TLE_N	0.04	4	0.37
PF00047	ig	PF00340	IL1	0.04	4	0.29
PF00047	ig	PF00531	Death	0.04	7	2.43

PF00047	ig	PF01839	FG-GAP	0.04	6	1.73
PF00059	Lectin_C	PF00089	Trypsin	0.04	5	0.94
PF00059	Lectin_C	PF00147	Fibrinogen_C	0.04	4	0.3
PF00069	Pkinase	PF01335	DED	0.04	8	3.3
PF00076	RRM_1	PF02037	SAP	0.04	5	0.89
PF00089	Trypsin	PF00207	A2M	0.04	4	0.35
PF00104	Hormone_recep	PF00096	zf-C2H2	0.04	9	4.23
PF00147	Fibrinogen_C	PF07645	EGF_CA	0.04	4	0.38
PF00249	Myb_DNA-binding	PF07716	bZIP_2	0.04	4	0.37
PF00320	GATA	PF00412	LIM	0.04	4	0.24
PF00386	C1q	PF01391	Collagen	0.04	4	0.26
PF00400	WD40	PF00400	WD40	0.04	5	0.95
PF00400	WD40	PF00515	TPR_1	0.04	5	0.98
PF00400	WD40	PF00856	SET	0.04	4	0.28
PF00439	Bromodomain	PF00641	zf-RanBP	0.04	4	0.37
PF00514	Arm	PF03810	IBN_N	0.04	4	0.33
PF00564	PB1	PF00595	PDZ	0.04	6	1.63
PF00595	PDZ	PF00858	ASC	0.04	4	0.28
PF00595	PDZ	PF02210	Laminin_G_2	0.04	6	1.79
PF00615	RGS	PF01462	LRRNT	0.04	4	0.35
PF00788	RA	PF07647	SAM_2	0.04	4	0.27
PF00928	Adap_comp_sub	PF07686	V-set	0.04	4	0.36
PF00012	HSP70	PF00397	WW	0.05	4	0.41
PF00014	Kunitz_BPTI	PF00089	Trypsin	0.05	4	0.39
PF00041	fn3	PF01839	FG-GAP	0.05	6	1.86
PF00071	Ras	PF00514	Arm	0.05	7	2.62
PF00097	zf-C3HC4	PF00531	Death	0.05	7	2.59
PF00097	zf-C3HC4	PF07714	Pkinase_Tyr	0.05	17	11.62
PF00100	Zona_pellucida	PF00069	Pkinase	0.05	5	1.12
PF00307	CH	PF00595	PDZ	0.05	9	4.31
PF00335	Tetraspannin	PF07686	V-set	0.05	4	0.39
PF00454	PI3_PI4_kinase	PF07714	Pkinase_Tyr	0.05	6	1.81
PF00531	Death	PF01454	MAGE	0.05	4	0.43
PF00627	UBA	PF00643	zf-B_box	0.05	4	0.4
