# Supplementary material for: Organization of Physical Interactomes as Uncovered by Network Schemas
Source: PLoS Comput Biol. 2008 Oct 24;4(10):e1000203. doi: 10.1371/journal.pcbi.1000203 (PMC2561054; doi:10.1371/journal.pcbi.1000203)
Supplement: Table S8 — a (top) gives the most frequent Pfam motifs in the filtered yeast interactome, along with the number of proteins they annotate. Table S8b (bottom) gives the Pfam motifs that take part in the most number of interactions in the filtered yeast interactome, computed as the sum of the degrees of all proteins annotated with the terms. (0.01 MB PDF) [file pcbi.1000203.s012.pdf]

Pfam ID	Name	Frequency in filtered yeast network
PF00069	Pkinase	94
PF00400	WD40	61
PF00271	Helicase_C	39
PF00076	RRM_1	34
PF07690	MFS_1	29
PF00004	AAA	28
PF00083	Sugar_tr	27
PF00172	Zn_clus	27
PF00270	DEAD	26
PF00071	Ras	24

Pfam ID	Name	Number of interactions in filtered yeast network
PF00069	Pkinase	285
PF00400	WD40	189
PF00004	AAA	157
PF00153	Mito_carr	126
PF00018	SH3_1	120
PF00076	RRM_1	120
PF00271	Helicase_C	115
PF00149	Metallophos	112
PF00172	Zn_clus	106
PB000002	Pfam-B_2	106
